# L1cam-mediated developmental processes of the nervous system are differentially regulated by proteolytic processing

**DOI:** 10.1038/s41598-019-39884-x

**Published:** 2019-03-06

**Authors:** Cecilie Linneberg, Christian Liebst Frisk Toft, Kasper Kjaer-Sorensen, Lisbeth S. Laursen

**Affiliations:** Department of Molecular Biology and Genetics, Aarhus University, Gustav Wieds Vej 10C, 8000 Aarhus C, Denmark

## Abstract

Normal brain development depends on tight temporal and spatial regulation of connections between cells. Mutations in L1cam, a member of the immunoglobulin (Ig) superfamily that mediate cell-cell contacts through homo- and heterophilic interactions, are associated with several developmental abnormalities of the nervous system, including mental retardation, limb spasticity, hydrocephalus, and corpus callosum aplasia. L1cam has been reported to be shed from the cell surface, but the significance of this during different phases of brain development is unknown. We here show that ADAM10-mediated shedding of L1cam is regulated by its fibronectin type III (FNIII) domains. Specifically, the third FNIII domain is important for maintaining a conformation where access to a membrane proximal cleavage site is restricted. To define the role of ADAM10/17/BACE1-mediated shedding of L1cam during brain development, we used a zebrafish model system. Knockdown of the zebrafish, *l1camb*, caused hydrocephalus, defects in axonal outgrowth, and myelination abnormalities. Rescue experiments with proteinase-resistant and soluble L1cam variants showed that proteolytic cleavage is not required for normal axonal outgrowth and development of the ventricular system. In contrast, metalloproteinase-mediated shedding is required for efficient myelination, and only specific fragments are able to mediate this stimulatory function of the shedded L1cam.

## Introduction

Development of the central nervous system depends on tightly regulated cellular connections facilitated by dynamic changes in interactions between adhesion molecules. One of these adhesion molecules, which plays a key role in the development of the nervous system, is a member of the immunoglobulin superfamily of cell adhesion molecules (Igcam), L1cam. Mutations in L1cam have been reported to be associated with numerous symptoms, including corpus callosum aplasia, mental retardation, adducted thumbs, spasticity of the upper and lower limbs, and hydrocephalus, collectively known as CRASH^1^ or L1 syndrome^2^. Accordingly, based on work in different model systems, L1cam has been described to be involved in diverse processes at different stages during development of the nervous system, including regulation of axonal outgrowth, fasciculation, formation of the ventricular system^3–5^, and myelination^6,7^.

Members of the L1 subfamily contain multiple extracellular domains, including 6 Ig domains and 5 fibronectin type III (FNIII) domains followed by a transmembrane and an intracellular domain. During brain development, L1cam mediates cell-cell adhesion by homo- or heterophilic interactions with other Ig superfamily members, or RGD-dependent and -independent binding to integrins^8^. L1cam has been reported to be shed from the cell surface of several different cell types, including neuronal cells^9^, and interestingly, the amount of proteolytic fragments in brain lysates peaks postnatally^10^, indicating a link between developmental changes and changes in L1cam shedding. Soluble variants containing different elements of the extracellular part of L1cam have *in vitro* been reported to induce neuronal outgrowth^11^, to enhance cell migration^10,11^, and to stimulate myelination^12,13^. Based on these experiments it has been suggested that soluble L1cam is shed from the cell surface and incorporated into the extracellular matrix to act as an attractant during cell migration or axonal outgrowth^9,14^. Alternatively, cleavage of L1cam may be required to allow dynamic changes in cell-cell adhesion^13^, or the released intracellular domain may have separate signaling functions^15,16^.

Several proteinases have been reported to be able to mediate cell surface cleavage of L1cam, including ADAM10, ADAM17, and BACE1^9,11,17,18^, and under some circumstances plasmin^19^ and myelin basic protein (MBP)^20^. Plasmin cleaves at two sites within the third FNIII domain following K842 or K845^19^, resulting in a soluble fragment of 140 kDa and an intracellular fragment of 80 kDa. In contrast, BACE1 has been reported to cleave L1cam between Y1086 and E1087, resulting in a soluble fragment of about 180 kDa, containing most of the extracellular domains including all of the Ig domains and the five FNIII domains^18^. The specific cleavage sites for ADAM10 and ADAM17 are unknown, and even though both proteinases display some preference for specific residues in the P1 and P1’ sites^21,22^, neither of these proteinases have a specific consensus sequence that allows prediction of substrate recognition and cleavage site. Based on the size of the proteolytic fragments, both ADAM10 and ADAM17 appear to cleave L1cam close to the BACE1 cleavage site adjacent to the transmembrane domain^17,18^.

Cell surface shedding of L1cam has been reported to be stimulated by PMA and pervanadate via different intracellular signaling pathways^23^, and dephosphorylation of the intracellular domain has been suggested to induce conformational changes that enhance shedding^24^. ADAM17 is known to be activated by PMA^25–27^, and accordingly, L1cam shedding can be enhanced by PMA stimulation^11^. The specific molecular mechanisms that potentially regulate shedding mediated by ADAM10 and BACE1 are, however, unknown. Furthermore, L1cam proteolysis has mainly been studied *in vitro* in mono cell cultures, and the functional properties during brain development of the different proteolytic fragments are unclear. How cell surface shedding of L1cam regulates cell-cell interactions during normal brain development is therefore an open question. We here aim to determine the role of L1cam cleavage *in vivo*, and to assess the involvement of proteolytic fragments in specific processes of brain development.

## Results

### Deletion of the two membrane-proximal FNIII domains provides proteolytic resistance to L1cam

**Figure 1:**
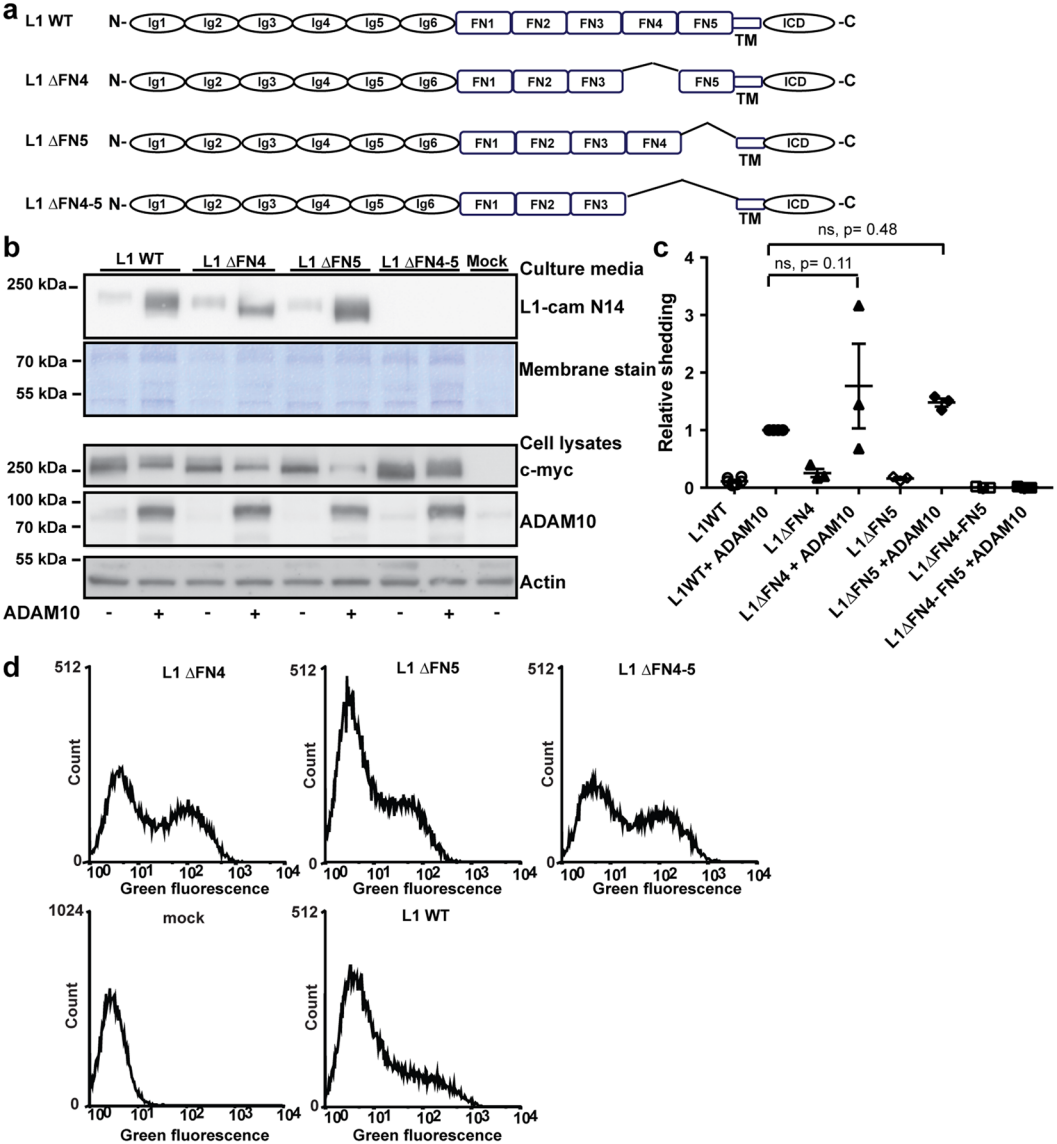
Deletion of the two membrane-proximal FNIII domains in L1cam is required to generate a proteinase resistant variant. (**a**) Diagram of WT L1cam and mutants with the two membrane proximal fibronectin type III (FN) domains deleted alone or in combination as indicated (**b**) HEK293T cells were transfected with plasmids encoding WT L1cam or deletion variants alone or in combination with ADAM10 as indicated. The extend of shedding of soluble L1cam into cell culture media was assessed by Western blotting using an antibody targeting the N-terminal of L1cam, membrane staining for total protein was used as loading control (2 top lanes). Expression of L1cam and ADAM10 in cell lysates was assessed by Western blotting, using antibodies targeting the C-terminal c-myc tag (L1cam), or ADAM10. Actin was used as loading control (3 bottom lanes). (**c**) The amount of soluble fragments in the culture medium was quantified and displayed relative to the amount of soluble fragments from cells co-transfected with WT-L1cam and ADAM10. Mean values +/− SEM for three independent experiments are plotted (Supplementary Fig. 11). Statistical significance was assessed by one-way ANOVA followed by Dunnett’s multiple comparison test. (**d**) HEK293T cells were transfected with plasmids encoding WT L1cam, deletion variants or an empty vector as indicated. The expression of the different variants on the cell surface was assessed by flow cytometry using an antibody targeting the N-terminal of L1cam. Example histograms are displayed. Mean fluorescence intensities for three independent experiments are displayed in Supplementary Fig. 1.

To assess the functional importance of L1cam proteolysis during brain development *in vivo*, a proteinase- resistant variant of L1cam is required. However, mutation of residues in the P1 and P1′ positions of the BACE1 cleavage site is not sufficient to abolish L1cam shedding *in vitro*^18^, and the precise cleavage site of ADAM10 is unknown, although based on the size of the released extracellular domain, cleavage occurs in close proximity to the plasma membrane. To attempt the generation of a proteinase-resistant L1cam variant, we constructed plasmids encoding variants in which individual FNIII domains in vicinity of the transmembrane domain were deleted (Fig. 1a). HEK293T cells were co-transfected with plasmids encoding the L1cam variants and ADAM10, and the amount of shedded L1cam fragments present in the culture media and the amount of full length proteins in cell lysates was assessed by Western blotting. Surprisingly, neither deletion of the 4th nor the 5th FNIII domain had any effect on ADAM10-mediated shedding (Fig. 1b,c), indicating that cleavage does not depend on a specific recognition motif in either of these modules. Interestingly, when both the 4th and 5th FNIII modules were deleted, no extracellular fragments could be detected in the culture media. The lack of surface shedding was not due to differences in the amount of protein expressed, as the total amount of L1cam in the cell lysates (Fig. 1b) and on the cell surface (Fig. 1d and Supplementary Fig. 1) was similar to wild-type protein. The inability of L1cam to undergo cleavage in the absence of FNIII4 and FNIII5 may suggest that modules FNIII1-3 are involved in the regulation of cell surface shedding. Previous biochemical analyses of the L1cam FNIII domains have suggested that they together form a globular structure. Specifically, interdomain interactions between FNIII1 and FNIII3 are thought to stabilize part of this structure^28^.

### Cell surface shedding of L1cam is regulated by conformational changes of the FNIII domains

**Figure 2:**
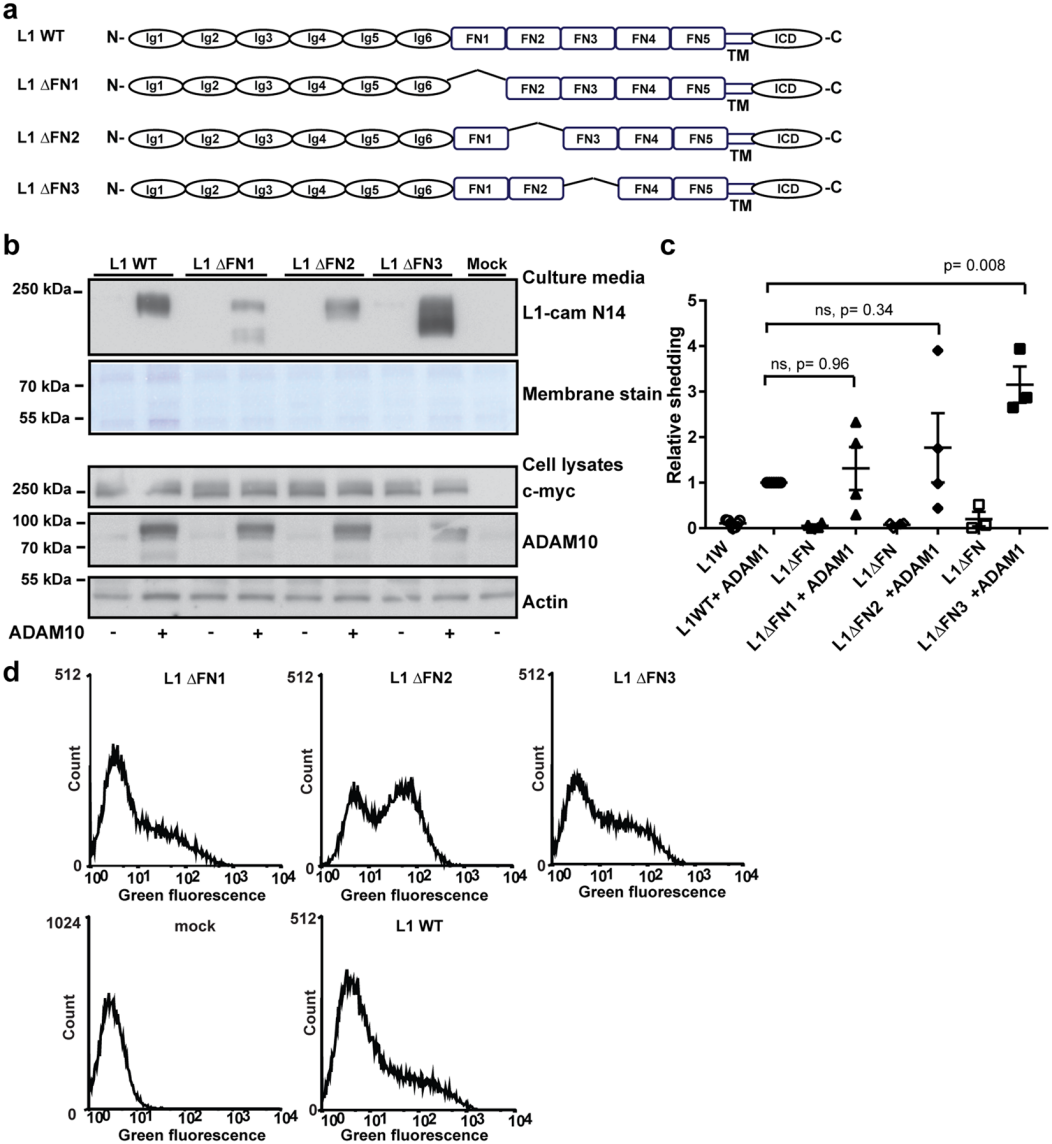
The first and third FNIII domain regulate ADAM10 mediated shedding of L1cam. (**a**) Diagram of WT L1cam and mutants with individual deletions of the first three fibronectin type III (FN) as indicated (**b**) HEK293T cells were transfected with plasmids encoding WT L1cam or deletion variants alone or in combination with ADAM10 as indicated. The extend of shedding of soluble L1cam into cell culture media was assessed by Western blotting using an antibody targeting the N-terminal of L1cam, membrane staining for total protein was used as loading control (2 top lanes). Expression of L1cam and ADAM10 in cell lysates was assessed by Western blotting, using antibodies targeting the C-terminal c-myc tag (L1cam), or ADAM10. Actin was used as loading control (3 bottom lanes). (**c**) The amount of shedded fragments in the culture medium was quantified and displayed relative to the amount of soluble fragments from cells co-transfected with WT-L1cam and ADAM10. For L1ΔFNIII1, both cleavage products were included for quantification. Mean values +/− SEM of at least three independent experiments are plotted (Supplementary Fig. 13). Statistical significance was assessed by one-way ANOVA followed by Dunnett’s multiple comparison test. (**d**) HEK293T cells were transfected with plasmids encoding WT L1cam, deletion variants or an empty vector as indicated. The expression of the different variants on the cell surface was assessed by flow cytometry using an antibody targeting the N-terminal of L1cam. Example histograms are displayed. Mean fluorescence intensities for three independent experiments are displayed in Supplementary Fig. 1.

**Figure 3:**
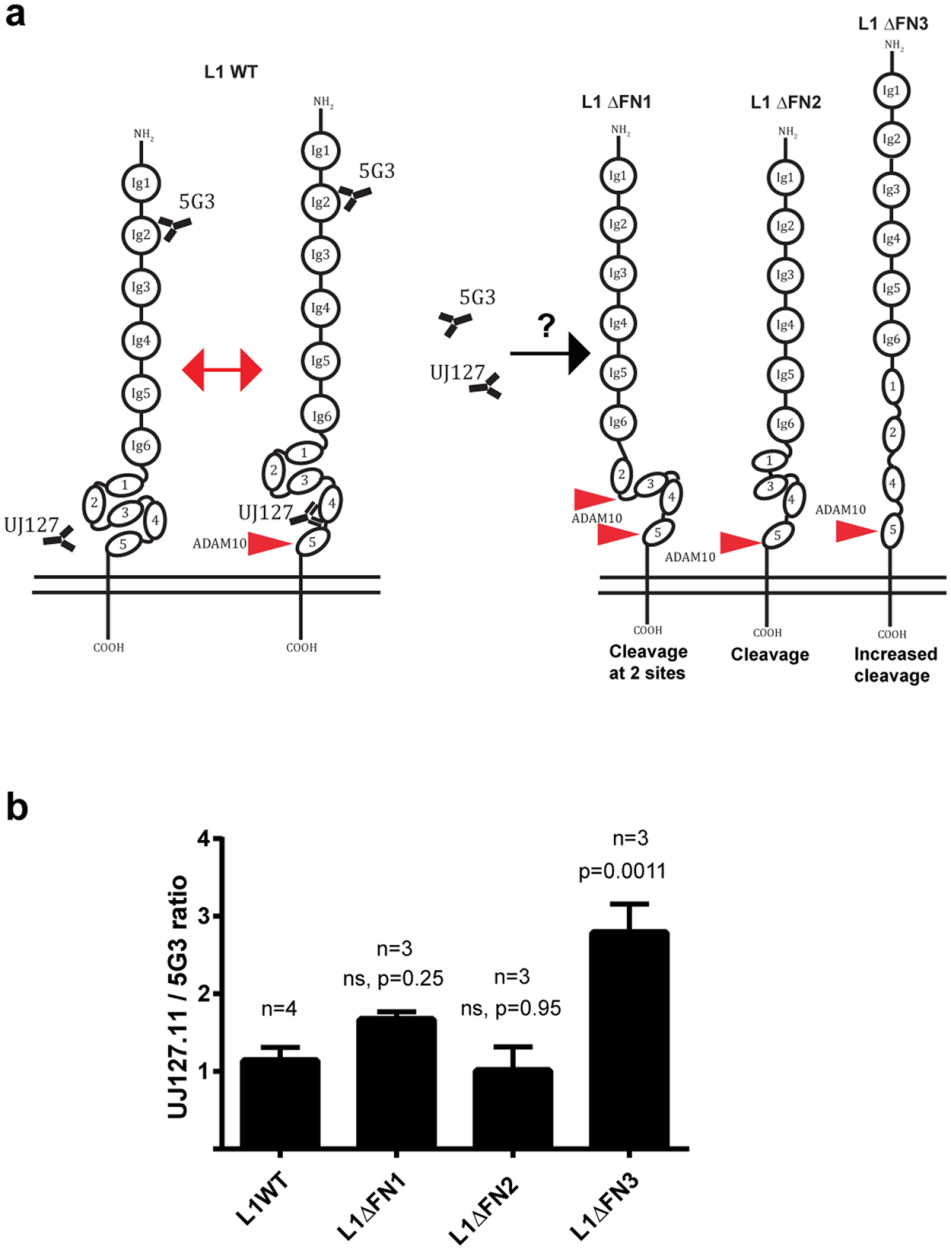
Deletion of the third FNIII domain increases the accessibility to the fourth FNIII domain. (**a**) Model for control of L1 proteolysis by conformational changes within the FNIII domains. (**b**) The accessibility of an antibody targeting the membrane proximal fourth FNIII domain for deletion mutants of L1cam in which the first three FNIII domains were deleted individually was analyzed by flow cytometry. The ratio of the mean fluorescence intensity for cells stained with an antibody targeting the fourth FNIII domain (UJ127-11) was given relative to the mean fluorescence intensity obtained for an antibody targeting the second Ig-domain in the N-terminal (5G3). Mean values +/− SEM for at least three independent experiments are plotted. Example histograms and dot-plots are displayed in Supplemental Fig. 4. Statistical significance was assessed by one-way ANOVA followed by Dunnett’s multiple comparison test.

To test if interactions between the first three FNIII domains are involved in stabilizing a proteinase-resistant conformation of the protein, these domains were also deleted individually (Fig. 2a), and the susceptibility towards ADAM10-mediated proteolysis was assessed (Fig. 2b–d and Supplementary Fig. 1). In the absence of FNIII1, an additional extracellular proteolytic fragment was generated, and in the absence of FNIII3, a marked increase in the amount of extracellular fragments was detected. No significant change was observed following deletion of FNIII2, however more of this variant is expressed at the cell surface so the cleavage efficiency may be slightly overestimated (Supplementary Fig. 1). These findings indicate that FNIII1 and FNIII3 are involved in controlling cleavage site accessibility. To assess the conformational change associated with regulation of access to the membrane proximal cleavage site, we determined binding of an antibody with an epitope in FNIII4, previously shown to display altered binding capacity following dephosphorylation of the intracellular domain^24^, relative to binding of an antibody recognizing an epitope in Ig2 distant from the cleavage site (Fig. 3a). An increased relative binding to FNIII4 was observed following deletion of FNIII3 (Fig. 3b), indicating that removal of this domain imposes a conformational change with increased access to the membrane proximal domains. Deletion of FNIII3-5 or all of the FNIII modules confirmed enhanced susceptibility to cleavage both when co-transfected with ADAM10 or when expressed in the absence of exogenous ADAM10 (Supplementary Fig. 2), further substantiating that the FNIII domains, in particular FNIII3, are required for controlled cell surface shedding of L1cam. This also suggests that substrate recognition and cleavage does not depend on the presence of a specific linear recognition motif. Interestingly, deletion of FNIII3 only resulted in enhanced cleavage by ADAM10, not by BACE1 or ADAM17. The latter however, required PMA stimulation of the cells to induce proteolysis, and, importantly, neither of these proteinases were able to cleave the variant in which both FNIII4 and FNIII5 had been deleted (Supplementary Fig. 3).

### L1camb knockdown results in early developmental abnormalities in the zebrafish brain

**Figure 4:**
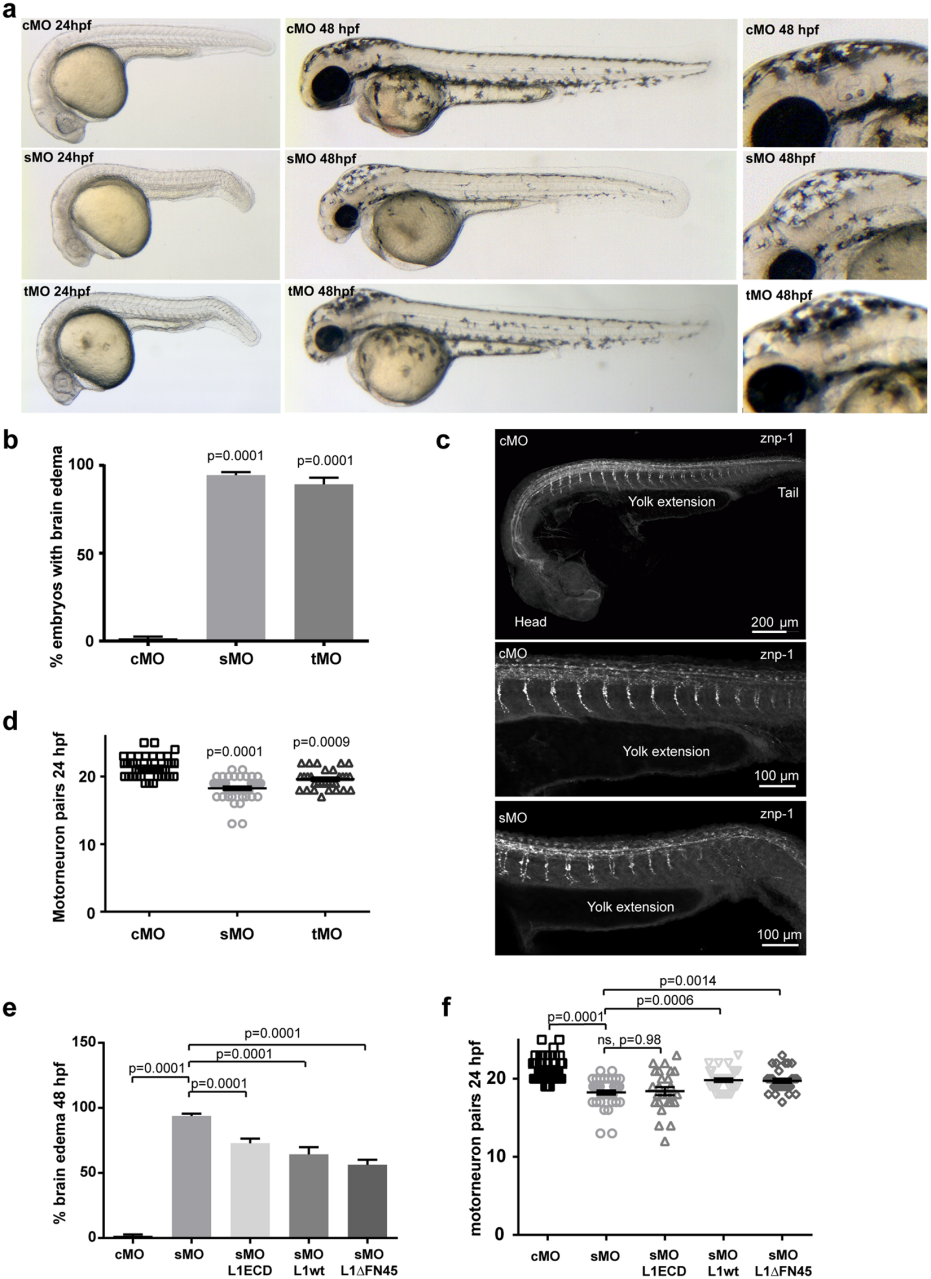
L1cam proteolysis is not required for axonal outgrowth from motor neurons, or development of the ventricular system. (**a**) Fertilized eggs from wild-type AB zebrafish, were injected with control morpholino (cMo), or morpholinos targeting splicing (sMo) or translation (tMo) of *l1camb*. The overall morphology of the larvae was assessed at 24 (left panel) and 48 (central panel) hpf. Enlarged view of the area of the mid- and hindbrain at 48 hpf (right panel). (**b**) Quantification of the penetration of the hydrocephalus phenotype, the percentage of larvae displaying an enlargement of the fourth ventricle was calculated for at least three independent injections. Mean values +/− SEM are plotted. The total number of larvae assessed in each group was cMo (149), sMo (215) and tMo (50). (**c**) Axonal outgrowth from motor neurons was assessed in fertilized embryos injected with morpholinos as in a, and following immunostaining for znp-1. (**d**) Quantification of axonal outgrowth from motor neurons. The number of motor neurons with visible axonal outgrowth at 24 hpf was counted. Mean values +/− SEM are displayed. Measurements are from at least three independent injections. (**e**) Fertilized eggs from wild-type AB zebrafish were injected with control morpholino (cMo) or a morpholino targeting splicing of *l1camb* alone or in combination with mRNA encoding wild-type (WT), proteinase-resistant (L1ΔFN45), or soluble (L1ECD) L1cam. The extend of hydrocephalus was evaluated at 48 hpf and the penetration of the hydrocephalus phenotype, the percentage of larvae displaying an enlargement of the fourth ventricle was calculated for at least three independent injections. Mean values +/− SEM are plotted. The total number of larvae assessed in each group was cMo (161), sMo (205), sMo- L1ECD (60), sMo-L1 WT (48), and sMo-L1ΔFN45 (51). Example pictures are displayed in Supplemental Fig. 6(f) Axonal outgrowth from motor neurons was assessed in fertilized embryos injected with morpholinos and mRNA as in e, following immune-staining with znp-1.The number of motor neurons with visible axonal outgrowth at 24 hpf was counted. Mean values +/− SEM are displayed. Measurements are from at least three independent injections. Statistical significance was assessed by one-way ANOVA followed by Dunnett’s multiple comparison test.

To assess the importance of cell surface shedding of L1cam *in vivo*, we used the zebrafish as a model system. The zebrafish expresses two orthologs to human L1cam, *l1cama* and *l1camb*, both predicted to have a domain organization similar to human L1cam, and importantly for use of the zebrafish as a model system, zebrafish ADAM10 also displays a high degree of similarity to the human protein, especially within the proteolytic domain (89%). Initially, the impact of the zebrafish paralog most widely expressed in the nervous system^29^, *l1camb*, on brain development was analyzed. Fertilized eggs from wild-type zebrafish were injected with control morpholinos or morpholinos targeting splicing or translation of *l1camb* mRNA. Following knockdown, the zebrafish larvae were assessed for phenotypes that previously have been linked to the L1 syndrome, or phenotypes observed in the knockout mice. These include development of hydrocephalus, changes in axonal outgrowth, and fasciculation defects. Knockdown of *l1camb* with both types of morpholinos resulted in a dramatic increase in the number of embryos with hydrocephalus at 48 hpf (Fig. 4a,b). In addition, impaired axonal outgrowth of primary motor neurons, assessed as the number of motor neuron pairs at 24 hpf, were observed (Fig. 4c,d). Furthermore, knockdown of *l1camb* also caused fasciculation abnormalities (Supplemental Fig. 5), in agreement with a previous report^30^. The importance of L1cam proteolysis at different developmental stages was assessed by analyzing the ability of wild-type, a noncleavable (*L1∆ FNIII4-5)*, or a soluble (*L1ECD)* variant to rescue the effect of knockdown on the development of brain edema and motor neuron outgrowth. All three L1cam variants were able to partially rescue the hydrocephalus phenotype (Fig. 4e), but interestingly, only the wild-type and noncleavable variant were able to rescue the axonal outgrowth of motor neurons (Fig. 4f). Combined, this suggests that shedding of L1cam is not required for any of these processes during early brain development. The inability of the soluble form of L1cam to rescue axonal outgrowth in motor neurons indicates that the presence of L1cam at the cell surface and potential signaling through the intracellular domain is important for this process. In contrast, the ability of the soluble variant to normalize the development of the ventricular system as efficient as the wild-type and proteinase-resistant L1cam variants suggest that signaling through the intracellular domain is not required for this process.

### Cell surface shedding of L1cam enhances myelination

**Figure 5:**
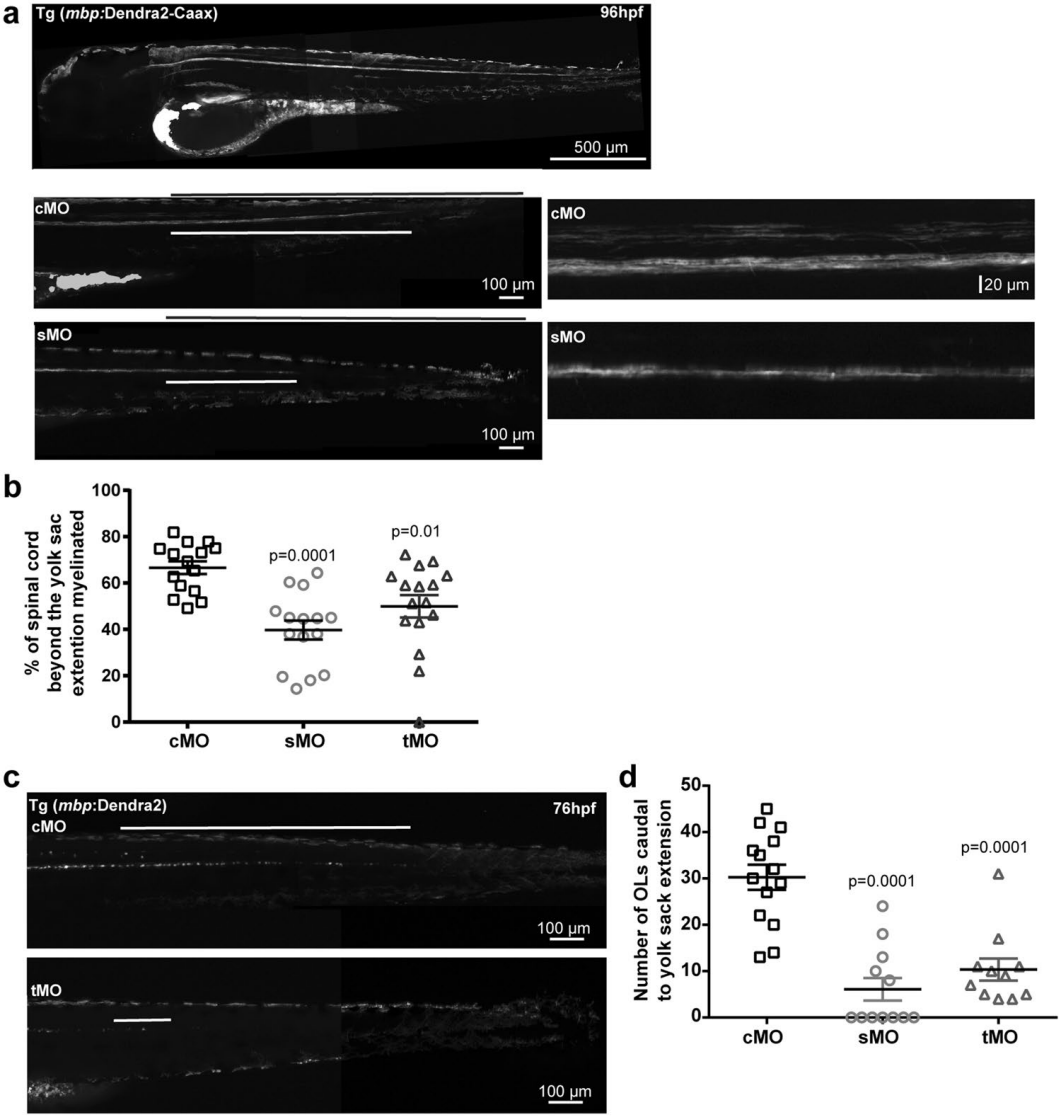
Knockdown of L1camb decreases the number of mature oligodendrocytes and inhibits myelination. (**a**) Fertilized eggs from *Tg(mbp:Dendra2-CAAX)* zebrafish, expressing a membrane targeted version of the fluorescent protein Dendra2 under control of the *mbpa* promoter, were injected with control morpholino (cMo), or morpholinos targeting splicing (sMo) or translation (tMo) of *l1camb*. The extent of myelination was assessed at 96 hpf. Micrographs of the posterior part of the fish are displayed (left panels) with the total length from the end of the yolk sac extension to the tail marked by the black line and the extend of myelinated fibers by the white line. Enlarged views of the area of the spinal cord surrounding the yolk sac extension for embryos injected with cMo, or sMo are displayed in the right panels. Note the reduced extent of myelinated fibers and the reduced myelination in both the ventral and dorsal tract of the spinal cord in larvae from *l1camb*_sMo injections compared to larvae from cMo injections. (**b**) The extent of myelinated fibers was quantified by measuring the percentage of the caudal spinal cord containing myelinated fibers and the mean values +/− SEM are plotted. The analyzed larvae were from at least three independent injections. (**c**) Fertilized eggs from a transgenic zebrafish line, Tg(mbp:Dendra2), expressing the fluorescent protein Dendra2 under control of the *mbpa* promoter, were injected with control morpholinos (cMo), morpholinos targeting splicing (sMo) or translation (tMo) of *l1camb*. The posterior part of the spinal cord caudal to the yolk sac extension at 76 hpf is displayed. (**d**) Quantification of the number of Dendra2-positive cells caudal to the yolk sac extension at 76 hpf. Mean values +/− SEM for analyzed larvae from at least three independent injections are plotted. Statistical significance was assessed by one-way ANOVA followed by Dunnett’s multiple comparison test.

In addition to phenotypes associated with early brain development, previous mouse^6^ and *in vitro* studies^13^ have indicated that L1cam may be important during the initiation of myelination. To assess the possible involvement of L1cam and L1cam proteolysis in myelination in the zebrafish, morpholino-mediated knockdown of L1camb was performed in a transgenic reporter line in which a membrane bound fluorescent protein was expressed under the *mbpa* promoter to allow direct visualization of myelin sheaths (Fig. 5a). Following knockdown, a clear reduction in the extent and number of myelinated fibers was observed compared to control larvae at 96 hpf (Fig. 5a). Quantification of the progression of myelination, expressed as the relative extent of myelination caudal to the yolk sac extension, confirmed a significant reduction in myelination (Fig. 5b). To test whether the reduced or delayed myelination was caused by a decrease in the number of mature oligodendrocytes or if it was due to an inability of the oligodendrocytes to efficiently ensheath axons, the experiment was repeated in a zebrafish line *Tg(mbp:Dendra2)* that allows quantification of the number of mature oligodendrocytes. Following knockdown, a marked reduction in the number of mature oligodendrocytes was observed in more than 80% of the analyzed larvae (Fig. 5c,d). This indicates that the absence of L1camb either impairs oligodendrocyte differentiation or enhances cell death of the newly generated oligodendrocytes. Such an increase in cell death may be caused by the absence of L1cam on the surface of axons, or it may be caused by an indirect effect on the neurons normally projecting their axons into the spinal cord. The organization of nerve fibers in the area around the yolk sac extension was therefore evaluated. In approximately 90% of the control larvae, there is a clear organization of nerve fibers into dorsal and ventral tracts in the analyzed area. In approximately 50% of the L1camb knockdown larvae, this organization was disturbed (Supplementary Fig. 6).

**Figure 6:**
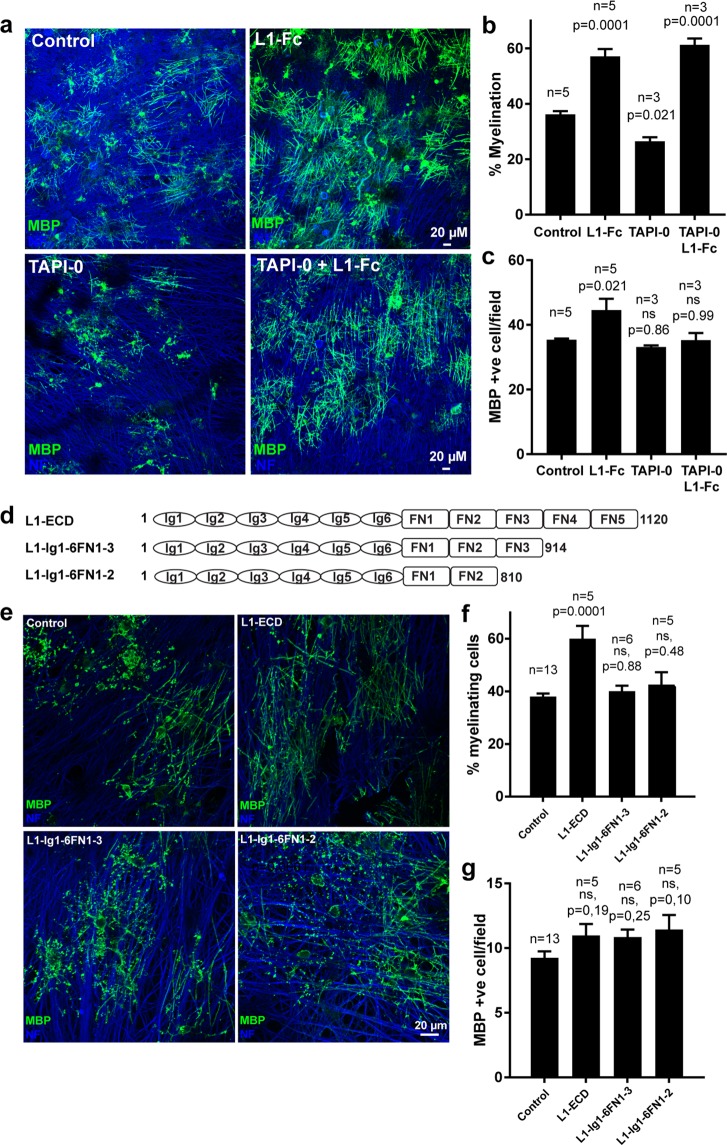
Metalloproteinase mediated cell surface shedding is required for efficient myelination. (**a**) Oligodendrocyte precursor cells (OPCs) were added to Dorsal Root Ganglion (DRG) neurons and co-cultured in the absence or presence of the metalloproteinase inhibitor TAPI-0 and/or soluble L1-Fc. The degree of myelination was analyzed following staining for MBP (marker of mature oligodendrocytes, green), and neurofilament (NF, neuronal marker, blue). Quantification is shown in the following two panels. Mean values +/− SEM are plotted (n values is the total number of coverslips analyzed in three independent culture sets). (**b**) Percentage of the MBP-positive cells forming at least two internode-like structures and therefore scored as myelinating. (**c**) The number of MBP-positive cells/10x field. (**d**) Schematic view of truncation variants of soluble L1. (**e**) OPCs and DRG neurons were co-cultured in the absence or presence of soluble L1 variants, L1-ECD, L1-Ig1-6FN1-3, or L1-Ig1-6FN1-4 as indicated. The degree of myelination was assessed following staining for MBP (green), and NF (blue). Quantification is shown in the following two panels. Mean values +/− SEM are plotted (n values is the total number of coverslips analyzed in three independent culture sets). (**f**) Percentage of the MBP-positive cells forming at least two internode-like structures and therefore scored as myelinating. (**g**) The number of MBP-positive cells/20x field. Statistical significance was assessed by one-way ANOVA followed by Dunnett’s multiple comparison test.

For further analysis of the effect of L1cam proteolysis on myelination, we switched to an alternative model system, a myelinating co-culture system of dorsal root ganglion (DRG) neurons and cortical oligodendrocyte precursor cells, in which the neurons were allowed to mature prior to any modification. The DRG neurons were differentiated for 3 weeks prior to the addition of oligodendrocyte precursors and co-cultured for 2 weeks in the presence or absence of the metalloproteinase inhibitor TAPI-0 and/or a soluble version of L1cam after which myelination was assessed following immunostaining for neurofilament and MBP (Fig. 6a). Under these conditions, a soluble variant of L1cam enhances myelination by increasing the percentage of mature MBP positive cells engaged in myelination. In contrast, the presence of the proteinase inhibitor resulted in a decrease in the percentage of mature MBP-positive cells engaged in myelination. Importantly, the decrease in myelination observed in the presence of the metalloproteinase inhibitor could be reversed by the addition of soluble L1cam, suggesting that cell surface shedding of L1cam indeed enhances the myelination efficiency (Fig. 6a–c).

Because L1cam fragments of variable length have been identified in brain lysates^10^, we asked whether the full length of the extracellular domain was required to stimulate myelination by successively truncating L1cam C-terminally (Fig. 6d). The variants were expressed in HEK293T cells and purified. Interestingly, only the longest variant containing all of the FNIII domains was able to enhance the percentage of mature oligodendrocytes engaged in myelination in the co-culture system (Fig. 6e–g), suggesting that L1cam-mediated stimulation of the myelination process depends on the specificity of the proteinase responsible for surface shedding.

## Discussion

Our analysis of ADAM10-mediated cleavage of L1cam suggests that cell surface shedding does not depend on a specific cleavage site within the membrane proximal region. Separate deletion of FNIII4 or FNIII5, the two membrane proximal FNIII domains, did not have any effect on proteolysis. However, the absence of both domains caused resistance to proteolysis. Interestingly, domains further away from the cleavage site also influenced proteolysis. Deletion of all of the FNIII domains, FNIII3-5, or FNIII3 alone resulted in a marked increase in ADAM10-mediated cleavage. Furthermore, additional analysis showed that deletion of the FNIII3 domain increased binding of an antibody with a conformational sensitive epitope in the FNIII4 domain, suggesting that cleavage in the membrane proximal region is regulated by conformational changes of the FNIII domains.

It is interesting to note that whereas the deletion of FNIII4-5 resulted in an L1cam variant showing resistance to cleavage by ADAM10, ADAM17 and BACE1, only ADAM10-mediated proteolysis was enhanced following the deletion of FNIII3. This indicates that L1cam cell surface shedding is regulated differently for these proteinases. A similar substrate-dependent difference in regulation of proteolysis by ADAM10 and ADAM17 has also been reported for cleavage of IL6R^31^. Additional ADAM10 substrates, which are regulated by conformational changes within their extracellular domains, include the Notch^32,33^ and ephrin^34^ receptors. In both cases, ligand binding to the substrate is required to permit ADAM-mediated proteolysis. In the Notch receptor, three LNR modules are involved in restricting access of the proteinase to the cleavage site, and forces applied by ligand binding and endocytosis into neighboring cells is suggested to induce the conformational changes required to allow access to the cleavage site^32,33^. The mechanism resulting in ligand-dependent cleavage of ephrin is slightly different. This has been suggested to involve cis-interaction between Eph and ADAM10, and conformational changes in the intracellular domain of Eph are suggested to facilitate ephrin cleavage in trans^35^. An alternative inside-out signaling mechanism has been suggested to regulate cleavage of Neuregulin-1 and CD44. In these cases, phosphorylation within the intracellular domain induces receptor dimerization, which again is suggested to lead to conformational changes in the extracellular domain, promoting access to the cleavage site^36–38^. Previous cell biological and biochemical studies of L1cam have suggested that changes in the phosphorylation status of the intracellular domain induce conformational changes and alter the extent of cell surface shedding^24^. Interestingly, the FNIII3 domain, here identified as important for regulation of access to the proteolytic site, has also been suggested to be involved in multimerization of L1cam and in integrin binding^28^. However, whether multimerization of L1cam is also directly involved in regulation of surface shedding remains to be tested. In other words, conformational changes in ADAM10 substrates have now been suggested in several independent cases and thus appear to be a general mechanism by which shedding is regulated.

To assess whether membrane shedding of L1cam is required during different stages of brain development, we determined the ability of a proteinase-resistant variant and a soluble variant to rescue phenotypes caused by L1cam knockdown in a zebrafish model system. Initially, we found that *l1camb* knockdown embryos show phenotypes similar to those previously reported for the L1cam knockout mice, and also similar to the abnormalities associated with L1 syndrome in humans. This includes development of hydrocephalus, aberrant axonal outgrowth, and fasciculation defects. Co-injection of morpholinos with mRNA encoding wild-type human L1cam rescued the hydrocephalus phenotype and normalized motor neuron development. Interestingly, mRNA encoding the proteinase-resistant variant rescued these phenotypes just as efficiently as the wild-type mRNA. In contrast, a soluble variant of L1cam, encoding only the extracellular domains, was sufficient to rescue the defects in ventricular development, but not axonal outgrowth of motor neurons. This indicates that the intracellular domain of L1cam is required for axonal outgrowth in these neurons. The ability of both the soluble and the proteinase resistant variants of L1cam to rescue ventricle abnormalities suggest that during this process, the extracellular part of L1cam is sufficient and L1cam may therefore mainly function as a ligand. Because of these differences, L1cam is likely to interact with different molecules during different stages of brain development. In some cases L1cam may function primarily as a ligand; in other cases, it mainly functions as a signaling or anchoring receptor. This is interesting with respect to the patients with mutations in L1cam, which appear to show variability in the severity of symptoms^39–41^.

Our observation that the proteinase resistant L1cam variant was just as efficient as the wild-type protein to rescue axonal outgrowth may be surprising based on the *in vitro* experiment showing that soluble L1cam has stimulatory effect on axonal outgrowth^42^. However, in these settings the cells still express L1cam on their surface. It is also interesting to note that in brain lysates, L1cam fragments, which result from membrane proximal cleavage, peaks postnatally^10^, and as such may primarily be required during synapse maturation and remodeling and during myelination, but not during migration of neuronal precursors and axonal outgrowth. An 80 kDa intracellular fragment, most likely resulting from cleavage in the third FNIII domain, is present during early development^10^. This fragment was recently suggested to be generated by the proteinase reelin, and was found to be able to rescue defects in migration of neuronal precursors during expansion of the cortical layers in the *reeler* mice, which lack reelin activity^43^. Altogether, this supports an important role for the intracellular domain of L1cam during early stages of brain development.

Following knockdown of *l1camb* in transgenic zebrafish, we observed a clear reduction in myelin sheath formation and a reduction in the number of mature oligodendrocytes. However, as it is clear that L1cam also affect axonal outgrowth and fasciculation, we cannot exclude that at least part of the reduction in myelin is caused by defects in axons, which have been shown to be required for survival of newly generated oligodendrocytes^44^. However, we only observed altered organization of axons in the dorsal and ventral tract of the spinal cord in approximately 50% percent of the L1camb knockdown embryos, but we observed a reduction in number of mature oligodendrocytes in approximately 80% of the analyzed larvae, suggesting that the effect of L1cam knockdown could also be a direct consequence of a reduced amount of L1cam. Such an effect would be in line with previous *in vitro* experiments, showing that L1cam enhances the survival of newly generated oligodendrocytes^13^.

In myelinating co-cultures, inhibition of ADAM-mediated proteolysis also reduced myelination. The addition of soluble L1cam reversed this effect, suggesting that shedding of L1cam is required for efficient myelination. Further analysis showed that the L1cam variants, which lack the FNIII4 and FNIII5 domains, were not sufficient to enhance myelination, indicating that fragments resulting from plasmin-mediated cleavage within FNIII3 cannot stimulate myelination. This is particularly interesting as an increase in plasminogen activator activity, observed e.g. during the inflammatory response in multiple sclerosis^45^, therefore is unlikely to have a stimulatory effect on CNS remyelination through generation of L1cam fragments, even though increased plasmin activity is suggested to enhance PNS remyelination^46^.

Altogether, our data suggest that shedding of L1cam is regulated by different mechanisms depending on the proteinase involved, that metalloproteinase-mediated shedding is only required during specific steps of brain development, and that the fragments generated by different proteinases have differential functions.

## Materials and Methods

### Plasmids for expression of recombinant proteins

Constructs encoding deletion mutations of L1cam were prepared by overlap extension PCR^47^ using the L1cam (NM_000425.4) inserted into the pcDNA3.1-myc-His vector via the *EcoRI* and *HindIII* sites as template. Sequences for the outer and inner primers are given in Supplementary Table 1.

Constructs encoding truncated soluble variants of L1cam were generate by PCR using the L1cam/pcDNA3.1-myc-His plasmid as template and inserted into pcDNA 3.1 myc-His (+) vector via the *EcoRI* and *HindIII* sites. The following primers were used; L1cam-ECD (5′-GTACGAATTCATGGTCGTGGCGCTGCGGTAC-3′ and 5′-GTAC AAGCTT CTCAGTGGCGAAGCCAGCAGGAGGG-3′), L1cam-ECDΔFN4-5 (5′-GTACGAATTC ATGGTCGTGGCGCTGCGGTAC-3′ and 5′-ACTGAAGCTT TGGGGTGCTGAAGGTGAACTC-3′) L1cam-ECDΔFN3-5 (5′-GTACGAATTC ATGGTCGTGGCGCTGCGGTAC-3′ and 5′-ACTGAAGCTT GTCCTCTCAGAGTAGCCGATAGTG-3′)

The plasmid encoding human-ADAM10 in a pRK5M vector was obtained from Addgene (plasmid#31717). A plasmid containing cDNA encoding human-BACE1 (NM_012104.4) inserted into pcDNA myc-his A(+) vector was obtained from Genscript. ADAM17 was obtained from Addgene.

### Transfection of HEK293T cells

Human embryonic kidney (HEK) 293T cells (293tsA1609neo) were maintained in high glucose DMEM supplemented with 10% fetal bovine serum, 2 mM glutamine, nonessential amino acids, and gentamicin (Thermo Scientific). For transient transfection, 3.0 × 10^6^ cells were plated onto 6-cm dishes and transfected 18 h later by calcium phosphate coprecipitation using 6 μg of plasmid DNA prepared by GenElute HP plasmid miniprep kit (Sigma). 9 hours post-transfection cells were transferred to serum-free medium (CD293, Thermo Scientific). Serum-free media were harvested 48 h post-transfection and cleared by centrifugation. Following media harvest cells were washed in phosphate buffered saline (PBS) and lyzed in ice-cold RIPA buffer (Sigma Aldrich) supplemented with proteinase and phosphatase inhibitors for 15 min on ice. Lysates were transferred to microtubes and cleared by centrifugation for 15 minutes at 4 °C.

### Protein purification

Purification of His-tagged recombinant proteins was carried out by affinity chromatography on a 1-ml HisTrap HP column (GE Healthcare). Serum-free media were diluted 1:1 in 20 mM NaH_2_PO_4_, 150 mM NaCl, pH 7.4, and loaded onto the column. The column was washed with 10 column volumes of 20 mM NaH_2_PO_4_, 1 M NaCl, pH 7.4, followed by 5 column volumes of 20 mM NaH_2_PO_4_ pH 5,5. The proteins were eluted with PBS supplemented with 10 mM EDTA, pH 7.4, and dialyzed against PBS, pH 7.4. Protein purity was assessed by SDS-PAGE, and quantification of purified proteins was done by amino acid analysis.

### Western blotting

To visualize proteins in serum free condition media (SFM), 200 µl SFM were precipitated with 1 mL 96% EtOH at −20 °C for 1 hour. Precipitated protein was isolated following centrifugation at 12.000 rpm, at 4 °C for 15 minutes and dissolved in sample buffer. Proteins in cell lysates or in SFM were separated by SDS-PAGE, and blotted onto a PVDF membrane (Millipore). The membranes were dried and blocked in 2% Tween-20, followed by overnight incubation with primary antibodies in 20 mM Tris, 150 mM NaCl, containing 0,1% FCS and 0.1% Tween-20, pH 7.6 (TBS-T) at room temperature. Membranes were washed in TBS-T and incubated for 1 hour with HRP-conjugated secondary antibodies (GE healthcare), washed again in TBS-T, and developed using ECL-Prime (GE Healthcare). Images were acquired with an Image Quant LAS 4000. The L1-N14 antibody was from Santa Cruz Biotechnology, the c-myc 9E10 clone was from DSHB, the β-actin antibody was from Sigma Aldrich and the ADAM-10 antibody (ab1997) was from Abcam.

### Measurement of L1cam proteolysis

ADAM10-, ADAM17- and BACE1-mediated cleavage of L1cam and L1cam mutants was analyzed following transient transfection of HEK293T cells. Fragments shedded into the culture media were precipitated as described above, separated by SDS-PAGE and analyzed by Western blotting using antibodies targeting the N-terminal of L1cam. Images were acquired with an Image Quant LAS 4000 (GE Healthcare) and band intensities were quantified using the Image Quant software. The relative cleavage of L1cam deletion variants were calculated by normalizing the amount of cleavage product to that of WT L1cam from the same transfection and analyzed within the same blot.

The level of endogenous expression of L1cam and ADAM10, compared to those introduced by transient transfection, is displayed in Supplementary Fig. 9. No endogenous expression of L1cam is detected and a marked increase in ADAM10 expression is observed following transient transfection.

### Flow cytometry

Transfected HEK293T cells were detached with phosphate-buffered saline (PBS) containing 5 mM EDTA 48 h post-transfection and washed with ice-cold DMEM medium (Gibco) containing 2% fetal calf serum (DMEM/FCS) and incubated on ice with primary antibody against L1cam (5G3 or UJ127-11, from Santa Cruz Biotechnology), at 10 μg/ml for 1 h. After washing three times in DMEM/FCS, the cells were incubated with Alexa-488 goat anti-mouse IgG (Thermo Scientific) diluted 1 to 500 for 60 min. After three washes, the cells were suspended in PBS with 2% paraformaldehyde and analyzed on a BD Biosciences flow cytometer. Approx. 10.000 cells were analyzed in each of at least three independent experiments.

### Cell culture

Primary oligodendrocyte precursor cells (OPCs) were obtained as described previously^48,49^. Briefly, dissociated rat neonatal cortices were cultured at 37 °C in 5.0% CO_2_ in Dulbecco’s modified Eagle’s medium (DMEM) with 10% fetal bovine serum (FCS) and penicillin/streptomycin in poly-D-lysine (PDL) coated flasks. By day 10, cultures consist of OPCs and microglia growing on an astrocyte monolayer (so-called mixed glia cultures). Cell populations enriched for OPCs were acquired by mechanically shaking them off the surface of the astrocytes and then removing microglia by differential adhesion on untreated petri dishes. All experiments involving isolation of rat tissue for primary culture were conducted according to Danish legislation and animals were euthanized following procedures approved by The Danish Animal Ethics Council prior to removal of tissue.

Myelinating co-cultures of oligodendrocytes and Dorsal Root Ganglion (DRG) neurons were generated as described previously^13,50^. In brief, embryonic DRG neurons were isolated from E15-E16 rats and dissociated with papain (1.2 U/mL; Sigma), L-cysteine (0.24 mg/mL; Sigma), and DNase I (0.40 mg/mL; Sigma) for 60 min at 37 °C. The dissociated cells were plated at a density of 200 × 10^3^ cells per coverslip (18 mm) coated with PDL and growth factor-reduced Matrigel (BD biosciences). The neurons were cultured for 17 days in DMEM (Gibco), 10% FCS (Gibco) in the presence of nerve growth factor (NGF) (100 ng/mL; Serotec). To remove contaminating cells, the cultures were pulsed three times for two days each with fluorodeoxyuridine (10 IU; Sigma) at day 2, 5, and 8 after seeding. After 17 days, the medium was changed to a 50:50 mixture of Sato’s modification of DMEM (Sato)^49,51^ and Neurobasal (Gibco), supplemented with 2% B27 (Gibco), N-acetyl cysteine (5 μg/mL; Sigma), and D-biotin (10 ng/mL), and 100.000 OPCs were added to each well. Fresh co-culture media supplemented with factors was added every third day. Proteinase inhibitor Tapi-0 (0,5 µM) or L1 variants at a final concentration of 1 µg/ml were used. After 18 days in co-culture, the cells were fixed with 4% PFA.

Following immunostaining with antibodies targeting, myelin basic protein (MBP) and neurofilament (NF), the degree of myelination was analyzed by fluorescent microscopy. For each tested condition coverslips from at least three independent experiments were analyzed. Only areas with at least 40% axonal coverage were analyzed. The total number of mature MBP positive oligodendrocytes was counted and each oligodendrocyte was scored as myelinating or nonmyelinating. Cells were scored as myelinating when at least two internode-like structures displaying smooth MBP staining covering an axon could be assigned to a cell body.

### Animal studies

Adult stocks of WT strain AB zebrafish and transgenic lines were maintained at 28.0 °C, 14-10 hour light-dark cycle on recirculating housing systems. Embryos were raised in E3 buffer (5 mm NaCl, 0.17 mm KCl, 0.33 mm CaCl_2_, 0.33 mm MgSO_4_, 10^−5^ (w/w) methylene blue, 2 mM HEPES, pH 7.4) at 28.5 °C. Embryos were sedated in tricaine (150 µg/ml) (Sigma-Aldrich) in E3 buffer when required. All experiments involving zebrafish were carried out according to Danish legislation and the fish were kept under protocol 2017-15-0202-00098 and 2017-15-0204-00018 approved by The Danish Animal Ethics Council.

To analyze the effect of L1cam knockdown on development of the nervous system morpholinos targeting *l1camb* were injected into fertilized eggs from WT AB zebrafish. Injections were performed at the 1–2 cell stage. The following morpholinos were used (Gene Tools): L1_camb tMo, (GCTGACTCTGCACTGGAGGCATTCT) (6 ng/embryo); L1_camb sMo(CCCAGCGAAAACTGTCAGTGAGAAA) (5 ng/embryo); As a control, the standard control oligo from Gene Tools was used. The knockdown efficiency with the splice-morpholino was assessed at 24, 48 and 72 hpf by RT-PCR, using the following primer sets: L1camb**_**forw, (5′-CCCATGCATACATTCAGATCCCAC-3′); L1camb rev, (5′-TGCTTCTTCACTTTGGCCAAGC) (Supplemental Fig. 6). At 24 hpf embryos were fixed in 4% PFA followed by immunostaining for znp-1 (DSHB) to assess motor neuron development. At 48 hpf live embryos were anesthetized with tricaine 150 µg/mL and imaged to assess the formation of brain edema.

Rescue experiments were performed to validate the specificity of the knockdown and to characterize the *in vivo* effect of the L1-mutants. mRNA encoding L1cam WT, L1camΔFN4-5 or L1cam ECD were generated from linearized plasmid using mMessage mMachine T7 Ultra Kit from Ambion and purified with RNeasy MinElute Cleanup Kit from Qiagen. Embryos at the 1–2 cell stage were injected with sMo and L1cam RNA (400 pg/embryo). The rescue effect was investigated by phenotyping of fixated embryos for motor neuron abnormalities at 24 hpf and living embryos for brain edema at 48 hpf as described above.

To assess the role of L1 during myelination, sMo (2,5 ng/embryo) and tMo (5 ng/embryo) morpholinos targeting L1 or cMo were injected into fertilized eggs obtained from transgenic zebrafish, *Tg(mbp:Dendra2)* or *Tg(mbp:Dendra2-CAAX)*. At 96 hpf the extend of myelination was evaluated in larvae from injections into *Tg(mbp:Dendra2-CAAX)* embryos by measuring the percentage of the spinal cord in which myelination was initiated. At 76 hpf the number of mature Dendra2-expressing oligodendrocytes caudal to the yolk sac extension was quantified in the *Tg(mbp:Dendra2)*. Only embryos with normal overall morphology were analyzed.

### Image acquisition and analysis

All fluorescent images were captured on a Zeiss Axio Observer.Z1 Apotome 2 microscope and image analysis and preparation were performed with Image J and Adobe Photoshop. For live imaging, transgenic zebrafish larvae were anesthetized with tricaine (150 µg/mL), embedded in 4% methyl cellulose with tricaine, and assessed by fluorescence microscopy using a 470 nm LED and a corresponding 52 HE (488/10 nm ex. 520/ 50 nm em) filterset. All images were taken from a lateral view of the spinal cord with anterior being left and dorsal top. A 10x objective was used.

### Statistical analysis

All statistical analyses were performed with the GraphPad Prism software. A fixed value of *p* < 0.05 was the criterion for reliable differences between groups. For multiple comparisons, one-way ANOVA was performed with relevant post-tests as indicated in the individual figure legends. Specific N-values are specified in the figure or figure legends whenever data are not plotted as individual data points. Data are presented as mean ± SEM.

## Supplementary information

Supplementary information

## Data Availability

All data generated or analyzed during this study are included in this published article (and its Supplementary Information Files).
