# Supplementary material for: L1cam-mediated developmental processes of the nervous system are differentially regulated by proteolytic processing
Source: Sci Rep. 2019 Mar 6;9:3716. doi: 10.1038/s41598-019-39884-x (PMC6403279; doi:10.1038/s41598-019-39884-x)

Supplementary material:

L1cam-mediated developmental processes of the nervous system are differentially regulated by proteolytic processing

*Cecilie Linneberg, Christian Liebst Frisk Toft, Kasper Kjaer-Sorensen, and Lisbeth S. Laursen**

Department of Molecular Biology and Genetics, Aarhus University
Gustav Wieds Vej 10C, 8000 Aarhus C, Denmark

Send correspondence to: Lisbeth S. Laursen, Department of Molecular Biology and Genetics, Aarhus University, Gustav Wieds Vej 10C, 8000 Aarhus C, Denmark. *Email: ll@mbg.au.dk*

Figure legends:

Supplementary figure 1: *Cell surface expression of L1cam-deletion mutants*

HEK293T cells were transfected with plasmids encoding WT L1cam or deletion variants, and the cell surface expression of the individual variants was assessed by flow cytometry using an antibody targeting the N-terminal. Mean values +/- SEM obtained by analysis of cells from 3 independent transfections are plotted.

Supplementary figure 2: *The FNIII domains negatively regulate cell surface shedding of L1cam*

(**a**) Model for control of L1cam proteolysis by conformational changes within the FNIII domains. Is the FNIII domains required to restrict proteolysis? (**b**) HEK293T cells were transfected with plasmids encoding WT L1cam or deletion variants alone or in combination with ADAM10 as indicated. The extend of shedding of soluble L1cam into cell culture media was assessed by Western blotting using an antibody targeting the N-terminal of L1cam, membrane staining for total protein was used as loading control (2 top lanes). Expression of L1cam and of ADAM10 in cell lysates was assessed by Western blotting, using antibodies targeting the C-terminal c-myc-tag (L1cam), or ADAM10. Actin was used as loading control (3 bottom lanes). (**c**) The amount of shedded fragments in the culture medium was quantified and displayed relative to the amount of soluble fragments from cells co-transfected with WT L1cam and ADAM10. Mean values +/- SEM of at least three independent experiments are plotted. Western blots used for quantification are displayed in Supplementary figure 15.Statistical significance was assessed by one-way ANOVA followed by Dunnett's multiple comparison test. (**d**) HEK293T cells were transfected with plasmids encoding WT L1cam, deletion variants or an empty vector as indicated. The expression of the different variants on the cell surface was assessed by flow cytometry using an antibody targeting the N-terminal of L1cam. Example histograms are displayed.

Supplementary figure 3: *Cell surface shedding of L1cam mediated by BACE1 and ADAM is not regulated by the third FNIII domain*

(**a**) HEK293T cells were transfected with plasmids encoding WT L1cam or deletion variants alone or in combination with ADAM10, BACE1 or ADAM17 in the absence of PMA as indicated. The extend of shedding of soluble L1cam into cell culture media was assessed by Western blotting using an antibody targeting the N-terminal of L1cam (top lanes). Expression of L1cam in cell lysates was assessed by Western blotting, using antibodies targeting the C-terminal c-myc tag (L1cam). Actin was used as loading control (center and bottom lanes). Displayed blots are from 2 different blots. Full size blots are shown in Supplementary figure 16. (**b**)

HEK293T cells were transfected with plasmids encoding WT L1cam or deletion variants alone or in combination with ADAM10, ADAM17 or BACE1 and the cells were stimulated with PMA as indicated. The extend of shedding of soluble L1cam into cell culture media was assessed by Western blotting using an antibody targeting the N-terminal of L1cam. Expression of L1cam in cell lysates was assessed by Western blotting, using antibodies targeting the C-terminal c-myc tag (L1cam). Actin was used as loading control (center and bottom lanes) Displayed blots are from 2 different blots. Full size blots are shown in Supplementary Figure 17. (**c**) The amount of shedded fragments in the culture medium following transfection with L1ΔFN3 and ADAM10, ADAM17+ PMA and BACE1 was quantified and displayed relative to the amount of soluble fragments from cells co-transfected with WT-L1cam and the respective proteinase. Mean values +/- SEM of at least three independent experiments are plotted (ADAM10 n=4, ADAM17 n=3, BACE n=4). One-sample t-test was used to determine if the relative shedding was different from 1.

Supplementary figure 4: Relative detection of L1cam variants by flowcytometry

Hek293T cells were transfected with L1cam-WT, L1camΔFNIII1, L1camΔFNIII2 or L1camΔFNIII3 and incubated with antibodies detecting the N-terminal (5G3, black and antibodies detecting an epitope in the membrane proximal region (UJ127.11, red) and subjected to flow cytometry.

Example dot-plot displaying gating used for all samples (top row) and example histograms used for calculation of relative antibody binding in Fig. 3 (second row). Corresponding dot-plots are show in the third row (5G3) and bottom row (UJ127.11).

Supplementary figure 5: *L1cam knockdown affects fasciculation*

(**a**) Fertilized eggs from wild-type AB zebrafish were injected with control morpholino (cMo), morpholinos targeting splicing (sMo) or morpholinos targeting translation (tMo) of *l1camb*. Fasciculation of axons of the Medial longitudinal fasciculus (MLF) were assessed at 20 hpf following staining with the neurofilament antibody 3A10. Enlarged view of part of the hindbrain at 20 hpf (cMo, top panel, sMo bottom panel). (**b**) Individual embryos were scored as normal or defasciculated and the distribution are displayed for cMo injected embryos (left columns), sMo injected (center columns) and tMo (right columns) as indicated. The total number of larvae assessed in each group after three independent injections was cMo (26), sMo (27) and tMo(25).

Supplementary figure 6: Rescue of the *l1camb* knockdown brain edema phenotype by L1cam variants

Example pictures for Fig 4e.

Fertilized eggs from wild-type AB zebrafish were injected with control morpholino (cMo) or a morpholino targeting splicing of *l1camb* alone or in combination with mRNA encoding wild-type (WT), proteinase-resistant (L1ΔFN45), or soluble (L1ECD) L1cam. The extend of hydrocephalus was evaluated at 48 hpf. The read lines (placed guided by the otoliths, arrows) are used to visualize the increase in size of the 4th ventricle following knockdown and the reduction in ventricle size following co-injection with the indicated *L1cam* mRNAs.

Supplementary figure 7: *L1 knockdown affects axonal organization in spinal cord tracts*
(**a**) Fertilized eggs from transgenic zebrafish Tg(mbpa:Dendra2-CAAX), were injected with control morpholino (cMo), or morpholinos targeting splicing (sMo) of *l1camb.* Organization of axons in the dorsal and ventral tract of the embryos was assessed at 72 hpf following staining with an antibody against acetylated tubulin. Enlarged view of the area of the spinal cord in the area just above the yolk sac extension at 72 hpf (cMo, top panel, sMo bottom panel). (**b**) Individual embryos were scored as normal or abnormal and the percentage with normal morphology is displayed as mean +/- SEM. The total number of larvae assessed in each group after three independent injections was cMo (25), sMo (22) and tMo (20).

Supplementary figure 8: *Knockdown of l1camb*

Fertilized eggs from wildtype AB zebrafish were injected with a control morpholino (cMo), or morpholino targeting splice sites of *l1camb* (sMo) as indicated. mRNA was isolated at 24, 48 and 72 hpf, and the level of splice alternations were assessed by RT-PCR.

Supplementary figure 9: Endogenous expression of L1cam and ADAM10 in HEK293 cells

HEK293T cells were left untransfected or transfected with plasmids encoding WT L1cam alone or in combination with ADAM10 as indicated. The extend of shedding of soluble L1cam into cell culture media was assessed by Western blotting using an antibody targeting the N-terminal of L1cam. Expression of L1cam and of ADAM10 in cell lysates was assessed by Western blotting, using an L1 specific antibody (UJ127.11) for detection of endogenous and exogenous L1cam. Actin was used as a loading control (2 center blots). An antibody targeting the C-terminal c-myc-tag was used to target exogenous, L1cam, and a ADAM10 specific antibodies was used to target endogenous and exogenous ADAM10. Actin was used as loading control (3 bottom blots).

Supplementary figure 10:

Full sized Western blots used for Fig. 1b

Supplementary figure 11:

Western blots used for quantification in Fig. 1c

Supplementary figure 12:

Full sized Western blots used for Fig. 2b

Supplementary figure 13:

Western blots used for quantification in Fig. 2c

Supplementary figure 14:

Full sized Western blots used for Supplementary figure 2b

Supplementary figure 15:

Western blots used for quantification in Supplementary figure 2c

Supplementary figure 16:

Full sized Western blots used for Supplementary figure 3a

Supplementary figure 17:

Full sized Western blots used for Supplementary figure 3b

Supplementary figure 18:

Full sized Western blots used for Supplementary figure 9

Supplementary table 1:

Outer primers	5′-GGTCACCTGGAGAATCAACGGGA-3′
	5′-AGTCAAGCTTTTCTAGGGCCACGGCAGGGT-3′.
Internal primers:	
L1camΔFN1	5′-TTCTTCTCTGGGGCTGCCACCGGCCCAGGGCTCC-3′
	5′-CCCTGGGCCGGTGGCAGCCCCAGAGAAGAACCCTG-3′
L1camΔFN2	5′-TCCAGCTCAGGGATTGCCTCTGGGGCTGCCTCAGGTGT-3′
	5′-TGAGGCAGCCCCAGAGGCAATCCCTGAGCTGGAAGGC-3′
L1CAMΔFN3	5′-CAGGCACTCCCTCTGGCTCTCCAGAGTAGCCGATAGTG-3′
	5′-CGGCTACTCTGGAGAGCCAGAGGGAGTGCCTGGCCA-3′
L1CAMΔFN4	5′-AAAATCTGAGATCCCAGACAAGGTGCTGAAGGTGAACTCGCTGG-3′
	5′-AGTTCACCTTCAGCACCTTGTCTGGGATCTCAGATTTTGGCAACATC-3′
L1CAMΔFN5	5′-GCCAGCAGGAGGGAGGGCCATAGTGCCTCCTTCCCGTA-3′
	5′-AAGGAGGCACTATGGCCCTCCCTCCTGCTGGCTTCGC-3′
L1CAMΔFN4-5	5′-AGCCGATGAACCAGCCCGCCTCGGGGTGGCCA-3′
	5′-CCACCCCGAGGCGGGCTGGTTCCATCGGCTTTGTGA-3
L1CAMΔFN3-5	5′-AGCCGATGAACCAGCCCAGCTCAGGGATTGCCTGGGG-3′
	5′-AGGCAATCCCTGAGCTGGGCTGGTTCATCGGCTTTGTGA-3′
L1CAMΔFN1-5	5′-AGTGGCGAAGCCAGCCCCAGGGCTCCCCACCACC-3′
	5′-GTGGGGAGCCCTGGGGCTGGCTTCGCCACTGAGGG-3′).

List of primers used to generate L1cam deletion variants.

Linneberg et al. Supplementary figure 1

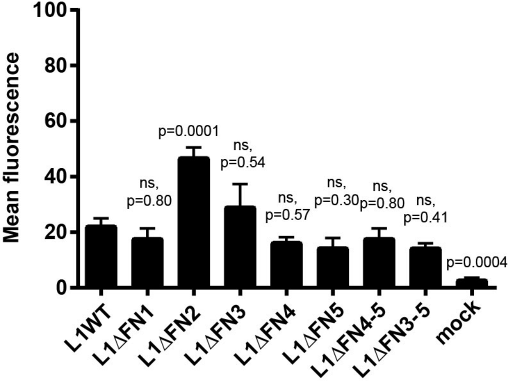

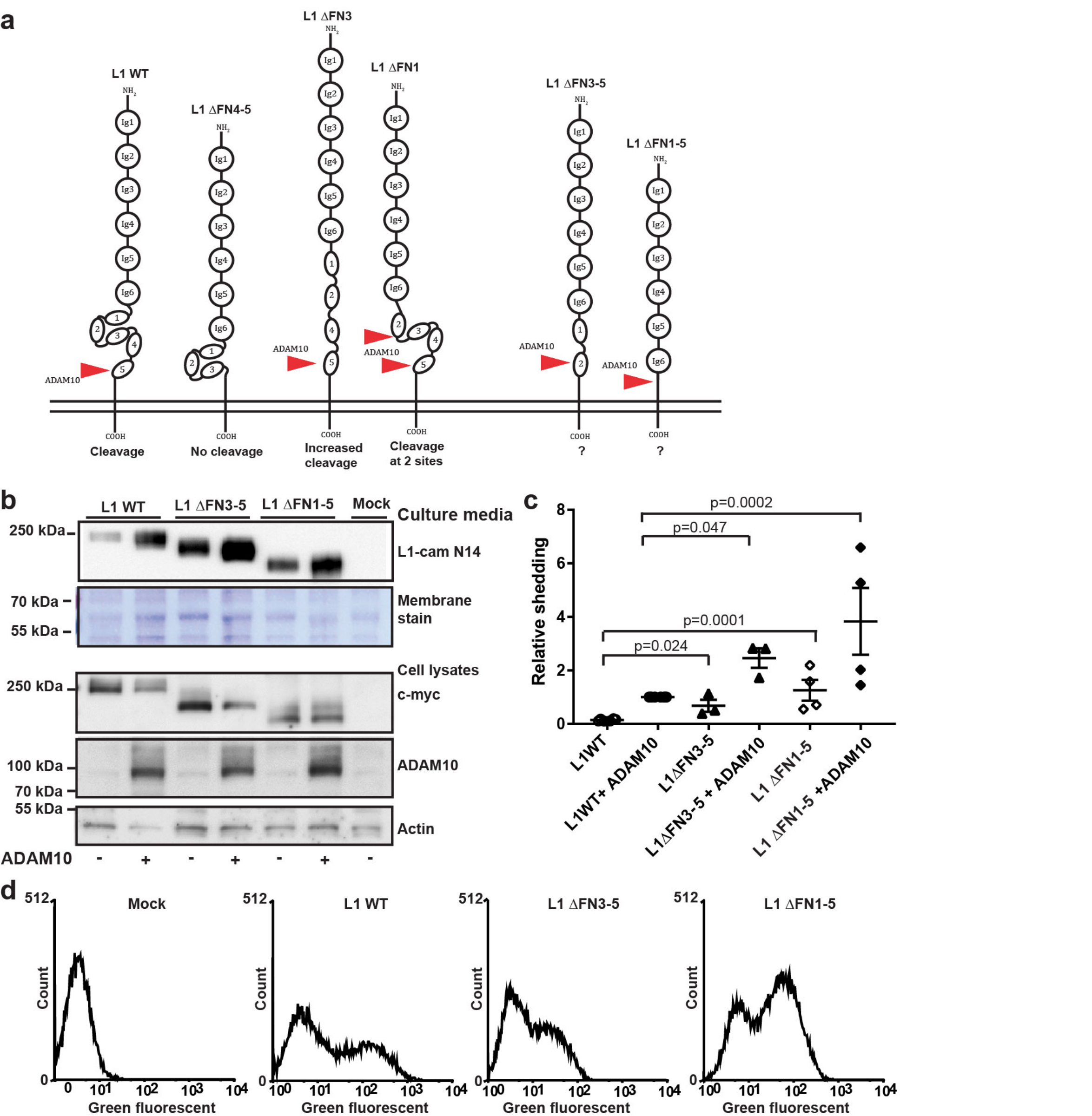
a
L1 WT
L1 ΔFN4-5
L1 ΔFN3
L1 ΔFN1
L1 ΔFN3-5
L1 ΔFN1-5
ADAM10
Cleavage
No cleavage
Increased cleavage
Cleavage at 2 sites
?
?
b
L1 WT
L1 ΔFN3-5
L1 ΔFN1-5
Mock
Culture media
L1-cam N14
Membrane stain
Cell lysates
c-myc
ADAM10
Actin
250 kDa
70 kDa
55 kDa
100 kDa
ADAM10 - + - + - + -
c
Relative shedding
p=0.0002
p=0.047
p=0.0001
p=0.024
L1WT
L1WT+ ADAM10
L1ΔFN3-5
L1ΔFN3-5 + ADAM10
L1 ΔFN1-5
L1 ΔFN1-5 +ADAM10
d
Mock
L1 WT
L1 ΔFN3-5
L1 ΔFN1-5
Count
Green fluorescent

Linneberg et al. Supplementary figure 3

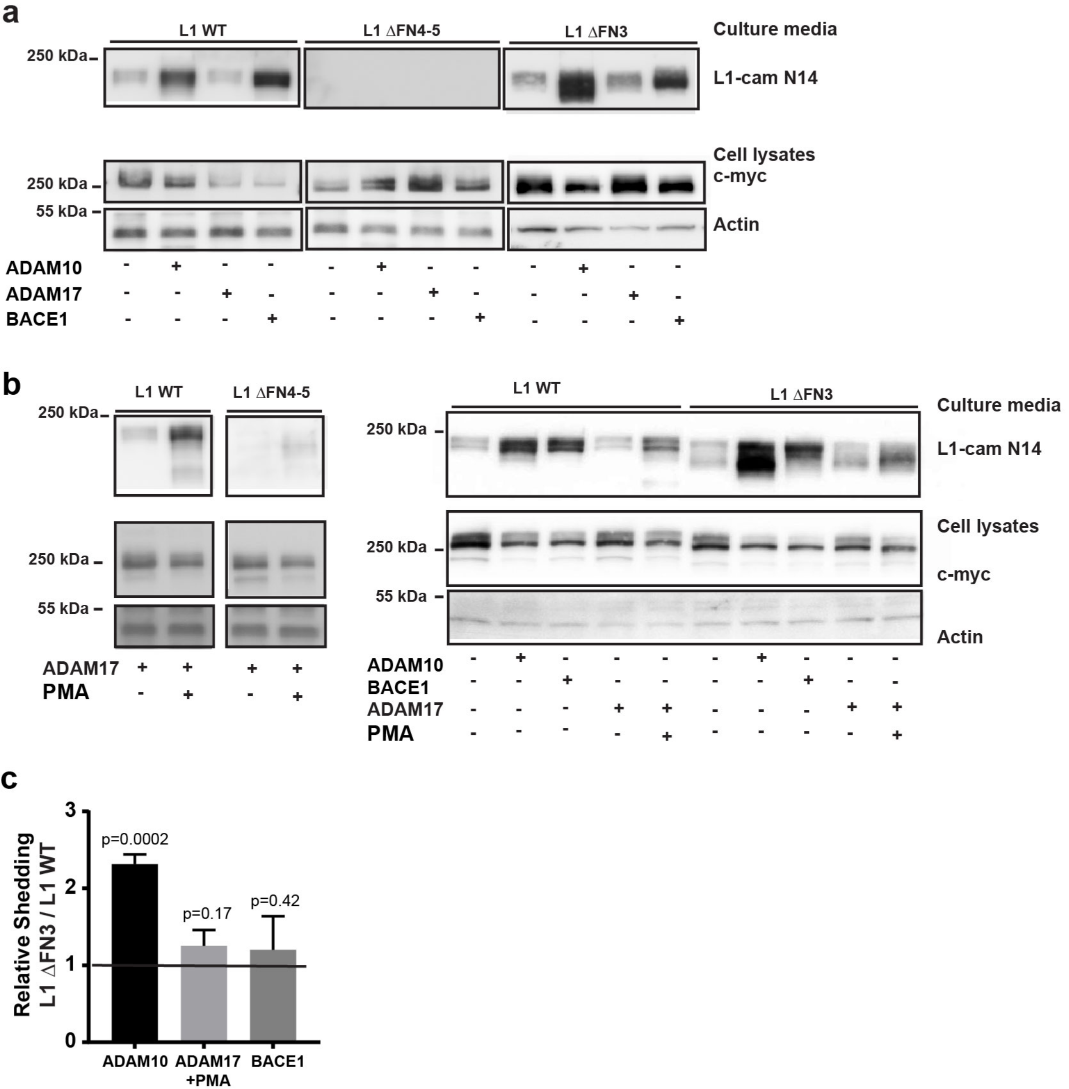

Linneberg et al. Supplementary figure 4

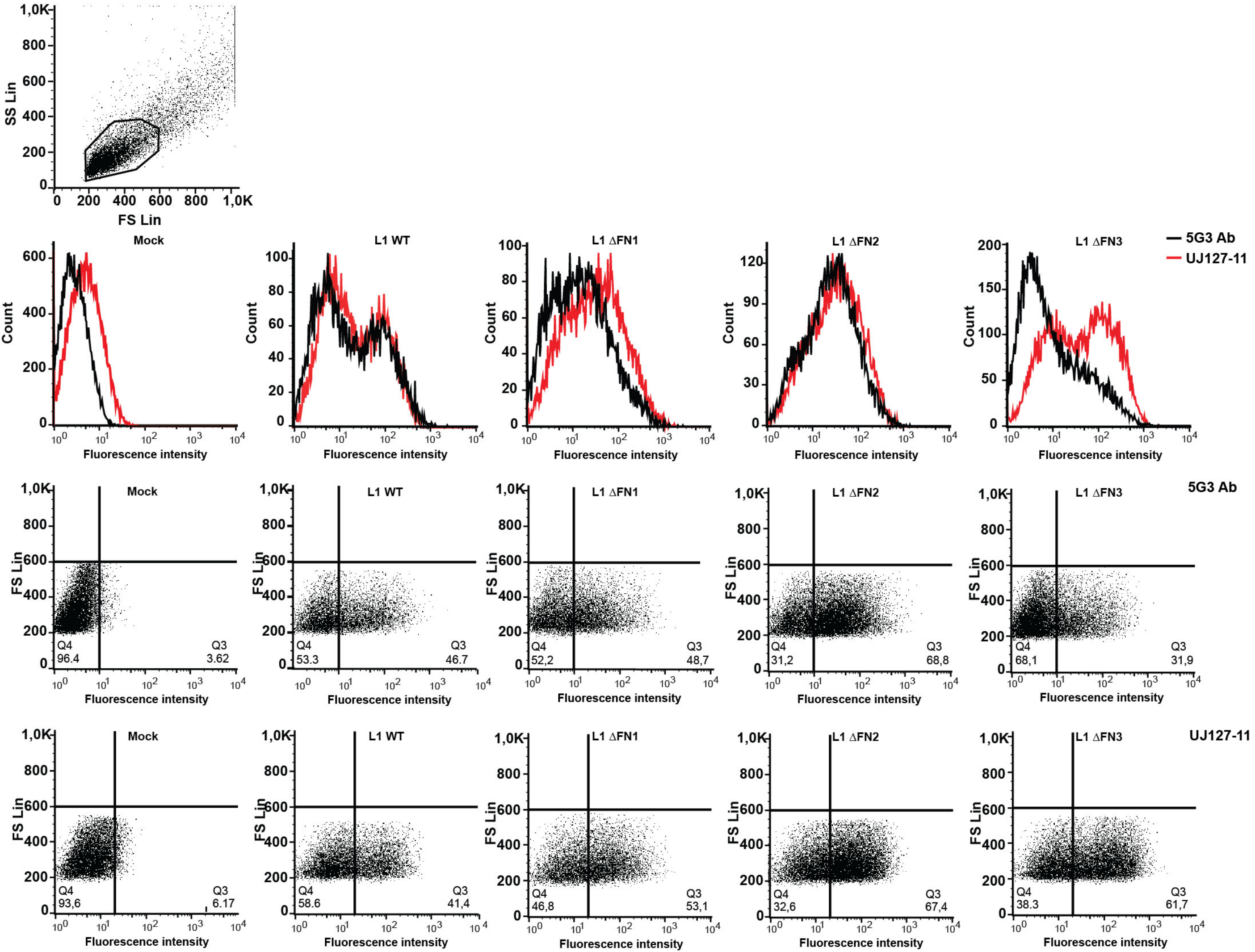

Linneberg et al. Supplementary figure 5

a

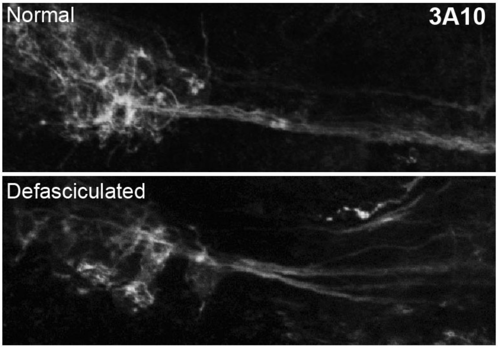

b

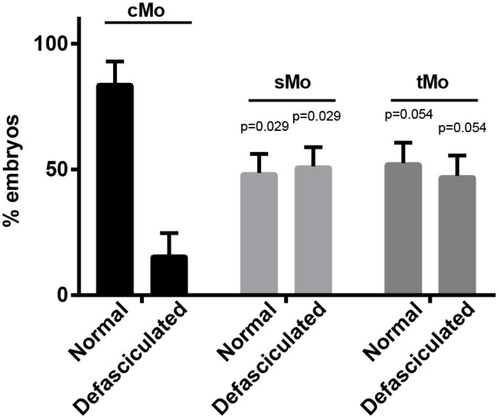

Linneberg et al. Supplementary figure 6

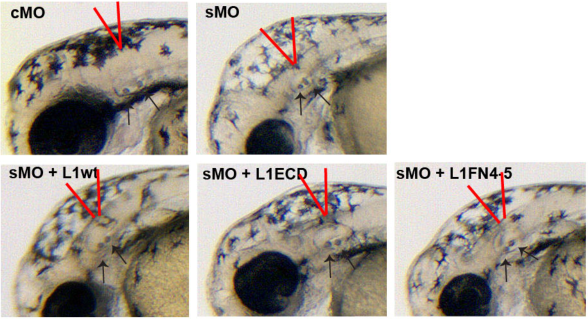

Linneberg et al. Supplementary figure 7

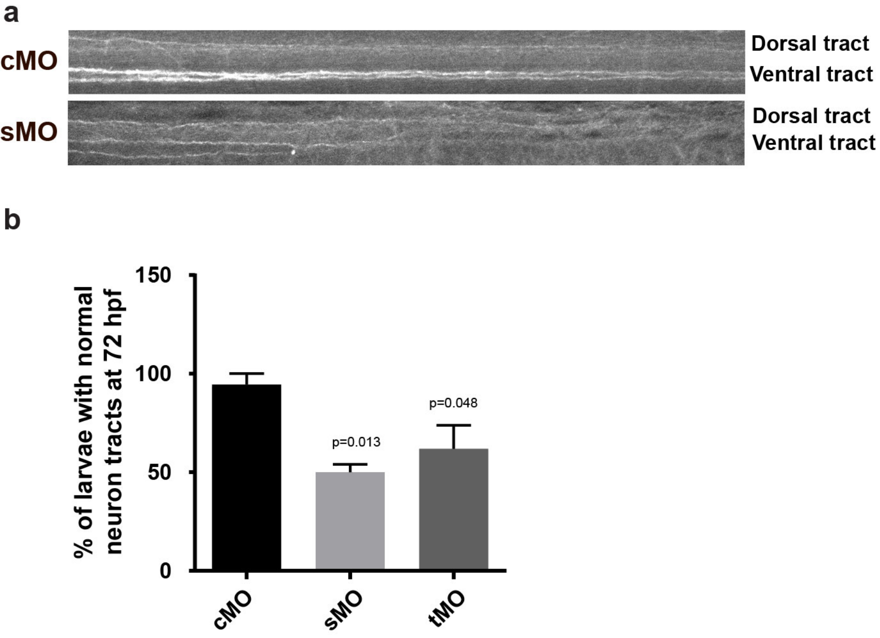

Linneberg et al. Supplementary figure 8

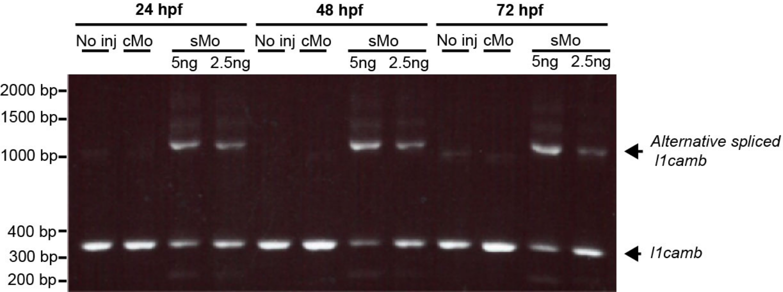

Linneberg et al. Supplementary figure 9

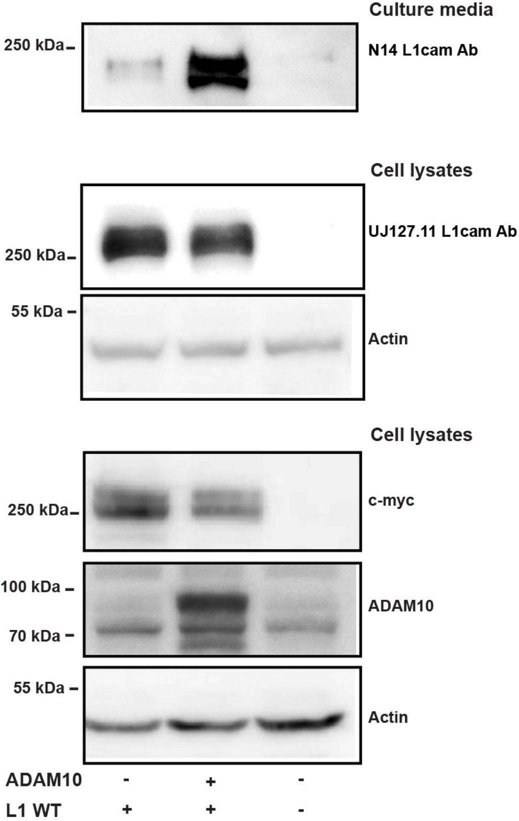

Full sized Western blots displayed in Figure 1b

Cell lysates

c-myc

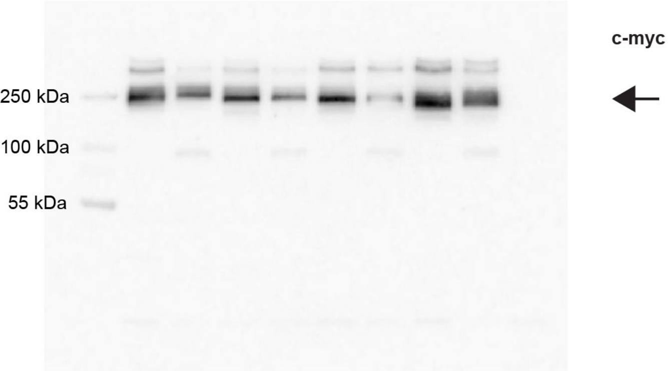

ADAM10

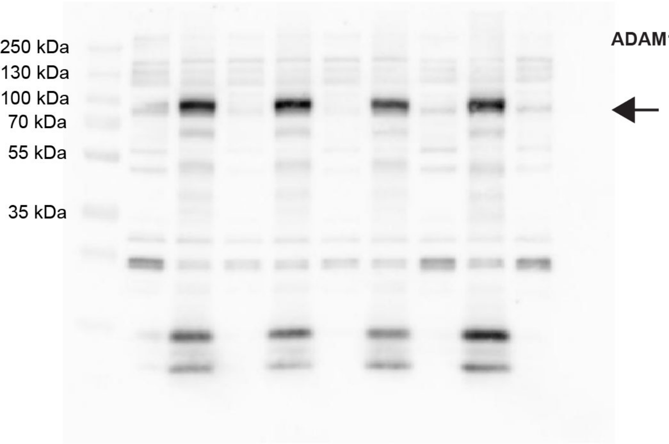

Actin

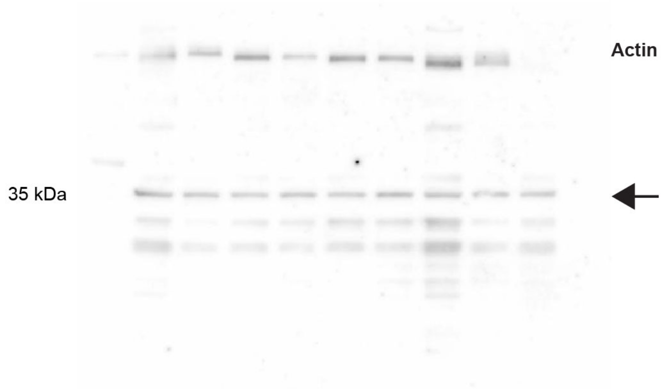

Culture media

L1-cam N14

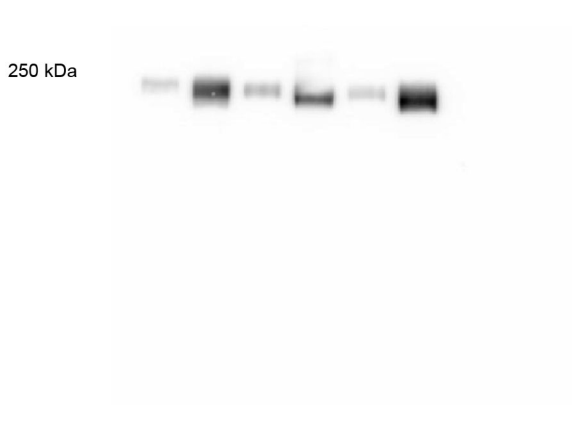

Membrane stain

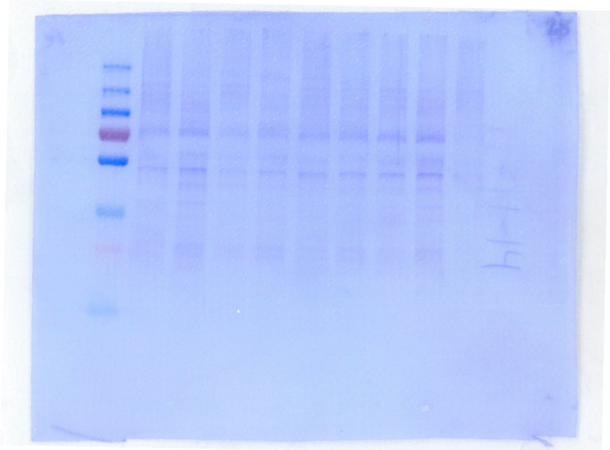

Western blots used for quantification of cleavage in figure 1c

Lanes not relavant for this figure

L1 WT | L1 ΔFN4 | L1 ΔFN5 | L1 ΔFN4-5

0,11 1 | 0,20 0,68 | 0,12 1,35 | 0,00 0,00 **relative cleavage**

\- + - + - + - + **ADAM10**

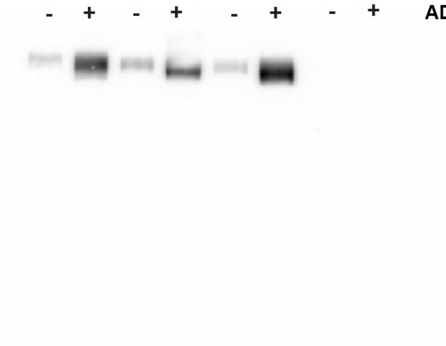

L1 WT | L1 ΔFN4-5

0,19 1 | 0,02 0,02 **relative cleavage**

\- + - + **ADAM10**

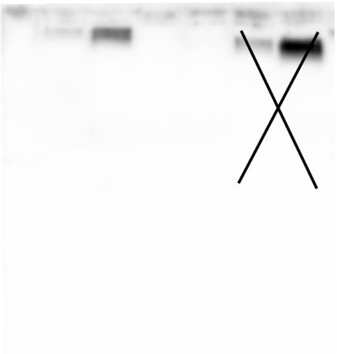

L1 WT | L1 ΔFN4 | L1 ΔFN5

0,17 1 | 0,40 3,17 | 0,19 1,58 **relative cleavage**

\- + - + - + **ADAM10**

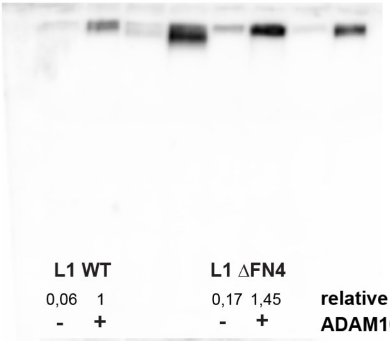

L1 WT | L1 ΔFN4-5

0,11 1 | 0,00 0,00 **relative cleavage**

\- + - + **ADAM10**

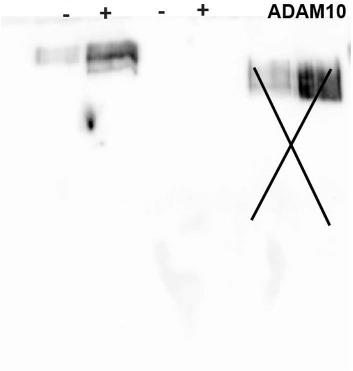

L1 WT | L1 ΔFN4

0,06 1 | 0,17 1,45 **relative cleavage**

\- + - + **ADAM10**

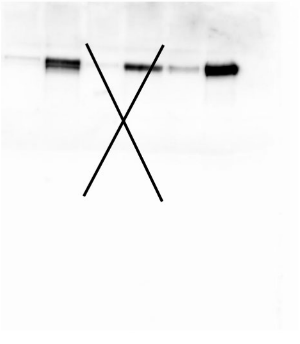

L1 WT | L1 ΔFN5

0,04 1 | 0,17 1,51 **relative cleavage**

\- + - + **ADAM10**

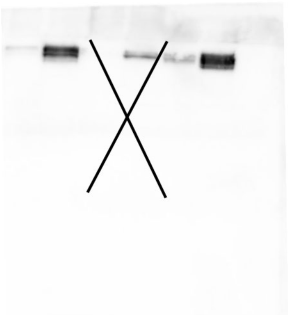

Full sized membrane for Western blots displayed in Figure 2b

Cell lysates

c-myc

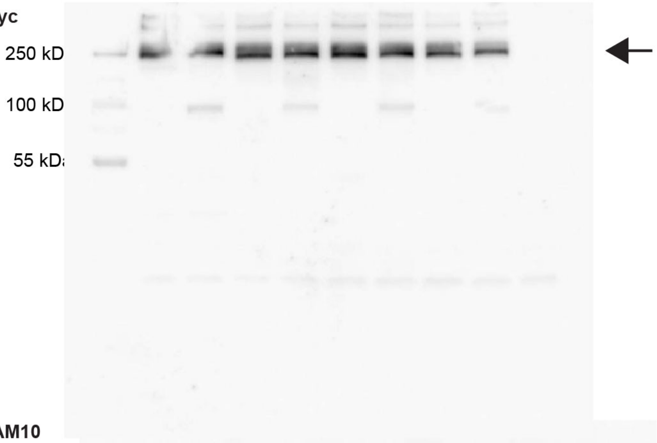

ADAM10

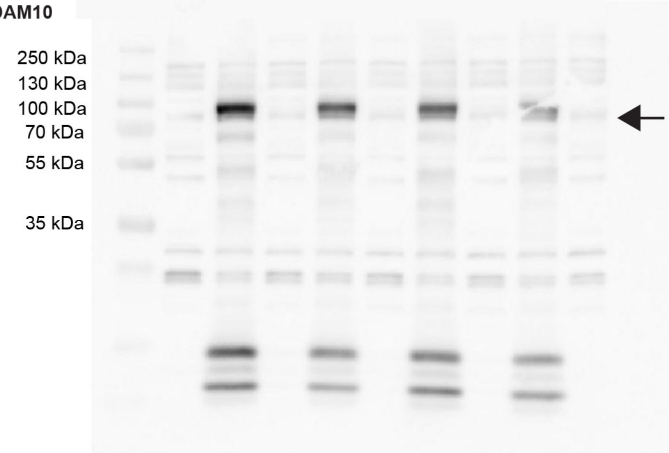

Actin

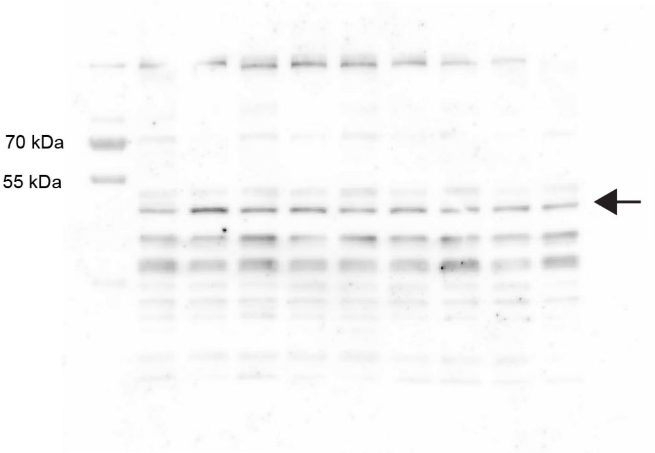

Culture media

L1-cam N14

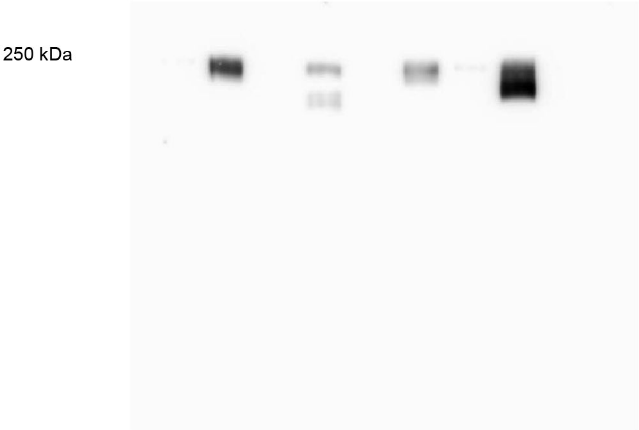

Membrane stain

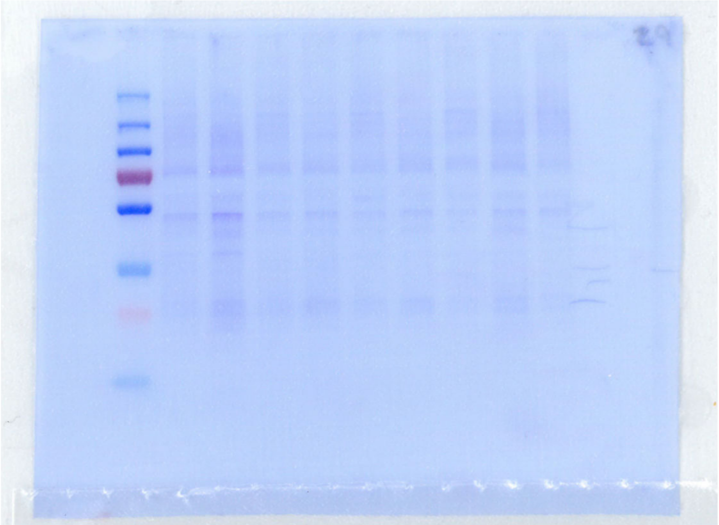

Linneberg et al. Supplementary figure 13

Western blots used for quantification of cleavage in Figure 2c

╳ **Lanes not relavant for this figure**

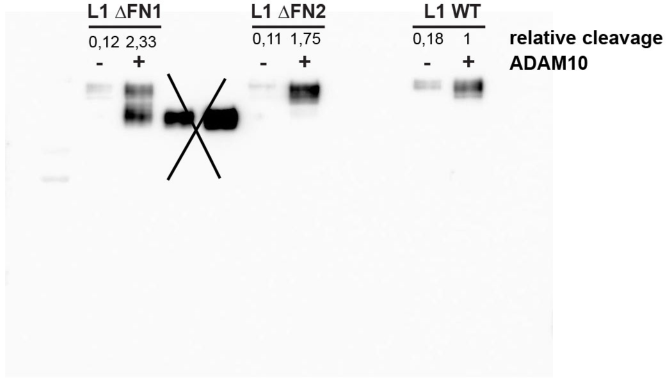

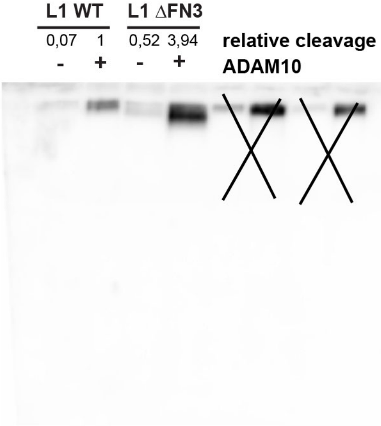

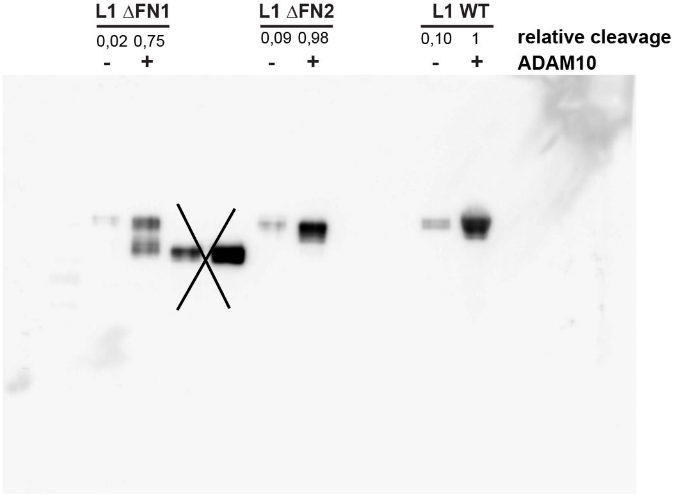

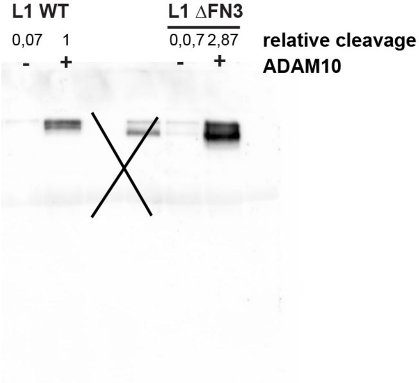

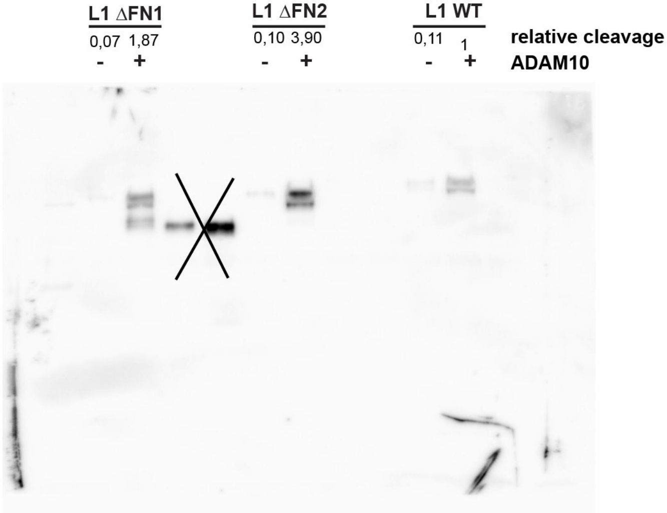

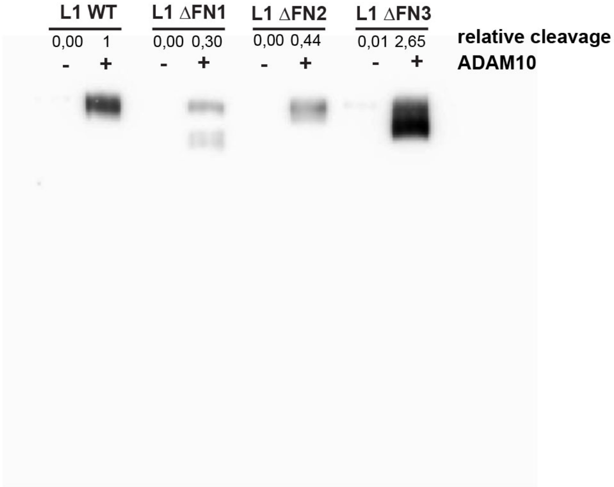

Full sized Western blots displayed in Supplementary figure 2b

Cell lysates

c-myc

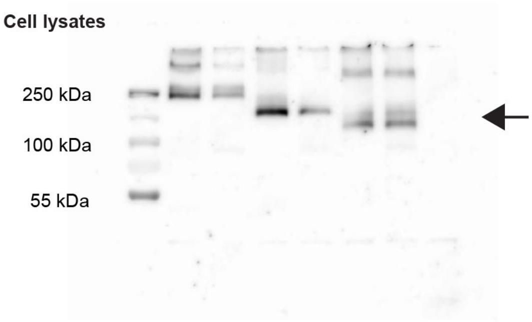

ADAM10

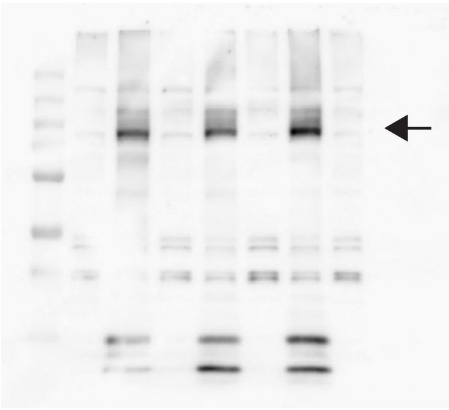

Actin

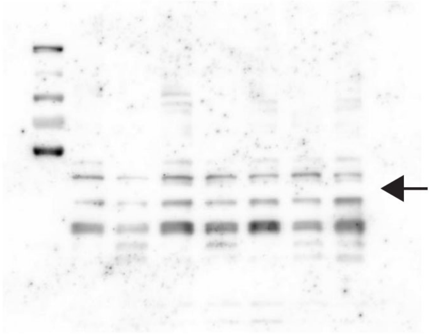

Culture media

L1-cam N14

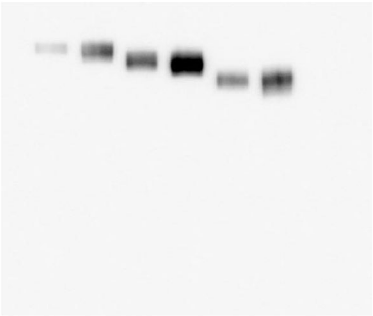

Membrane stain

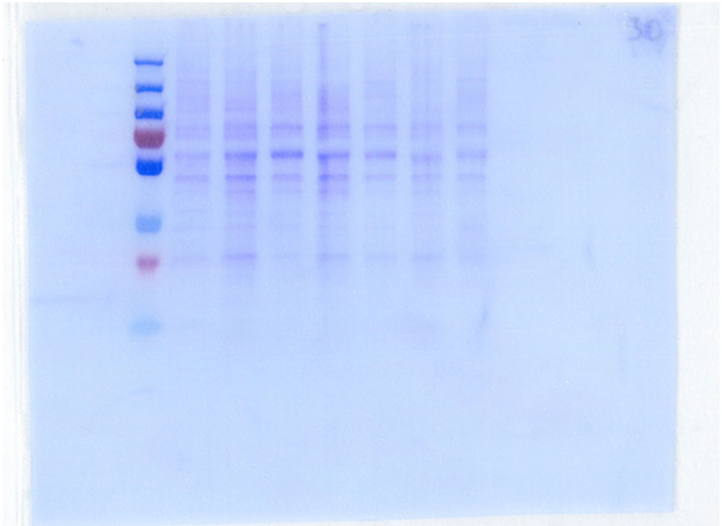

Linneberg et al. Supplementary figure 15

Western Blots used for quantification of ADAM10 mediated cleavage in Supplementary figure 2c

X Lanes not relavant for this figure

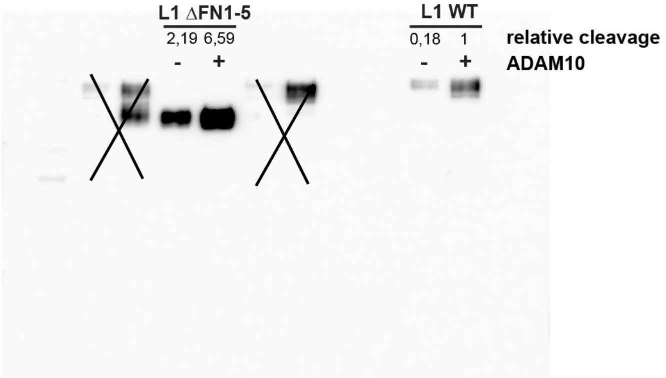

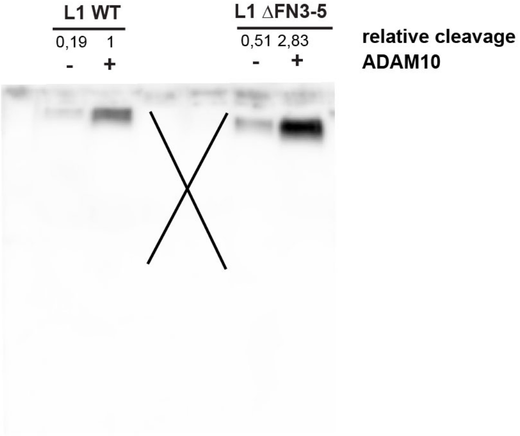

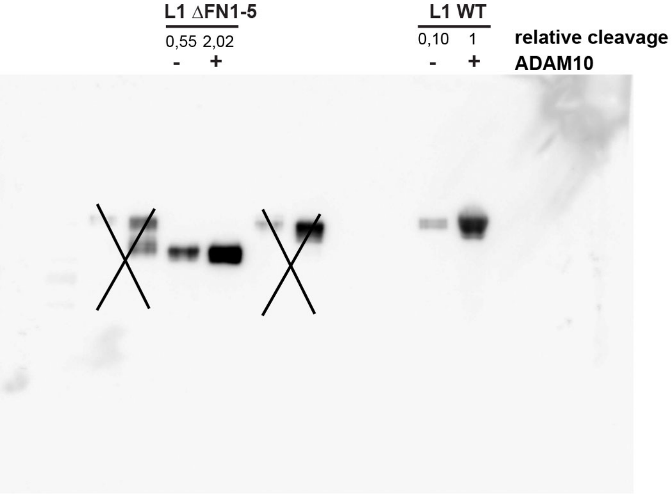

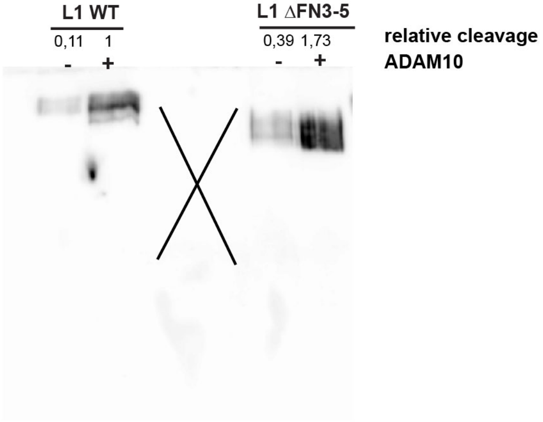

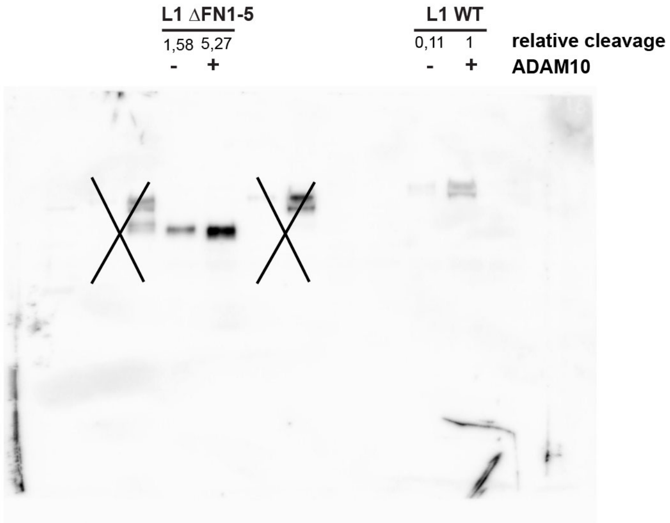

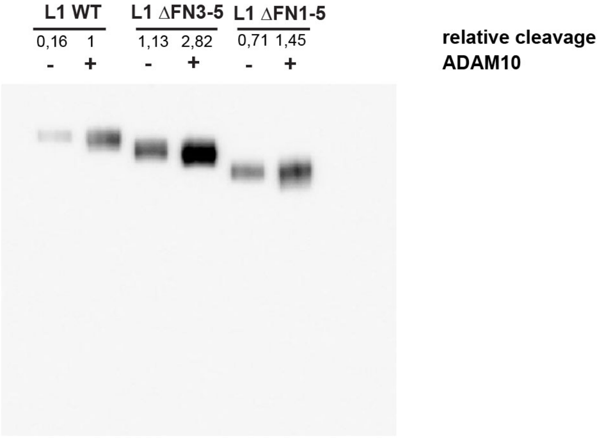

Full sized Western blots displayed in supplementary figure 3a

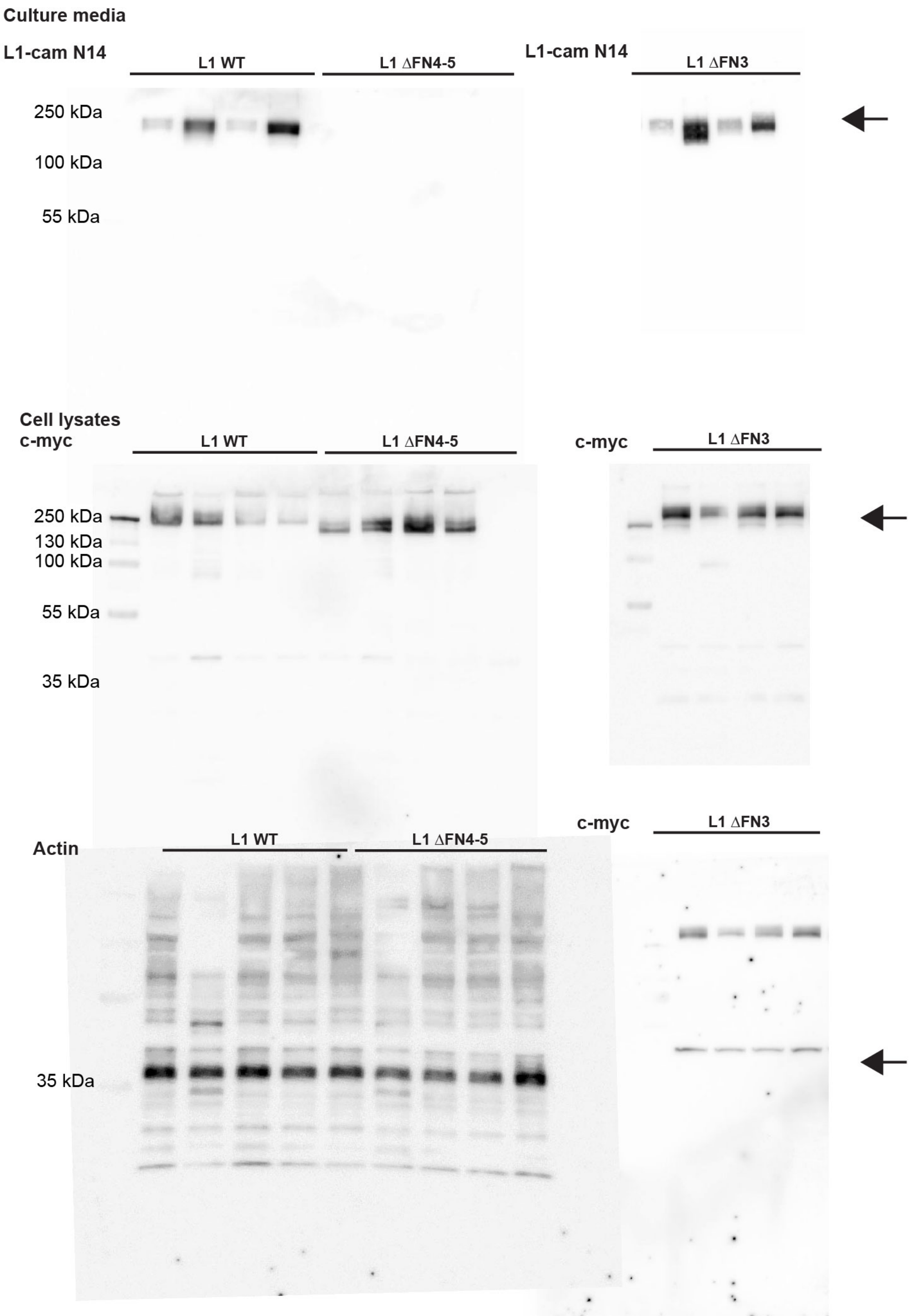

Full sized Western blots displayed in supplementary figure 3b

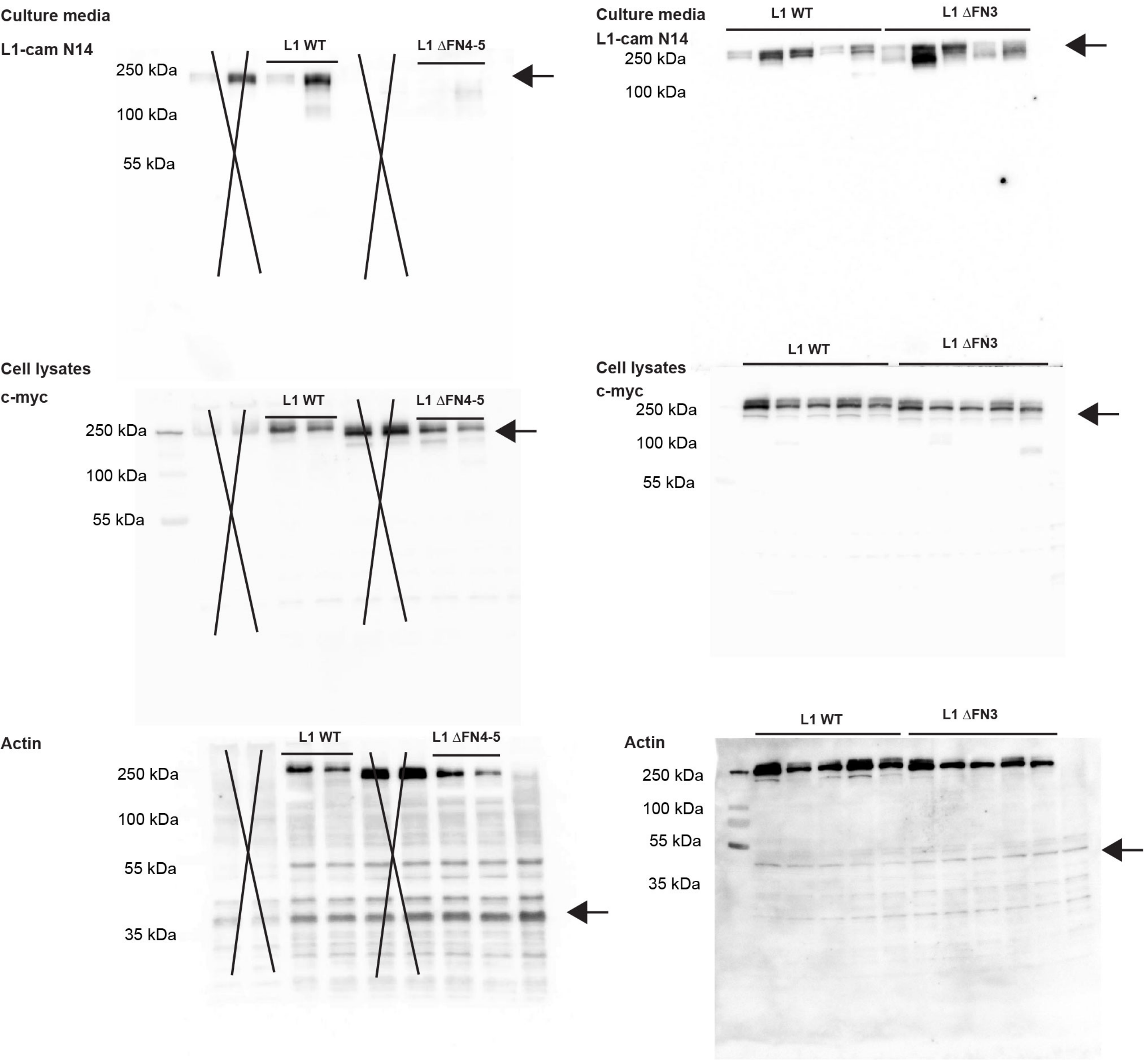

Full sized Western blots displayed in supplementary figure 9

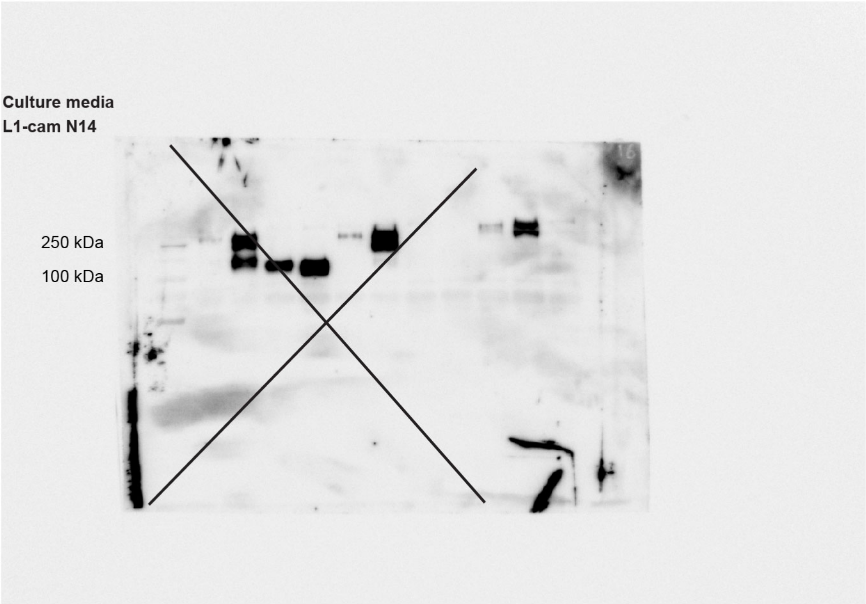

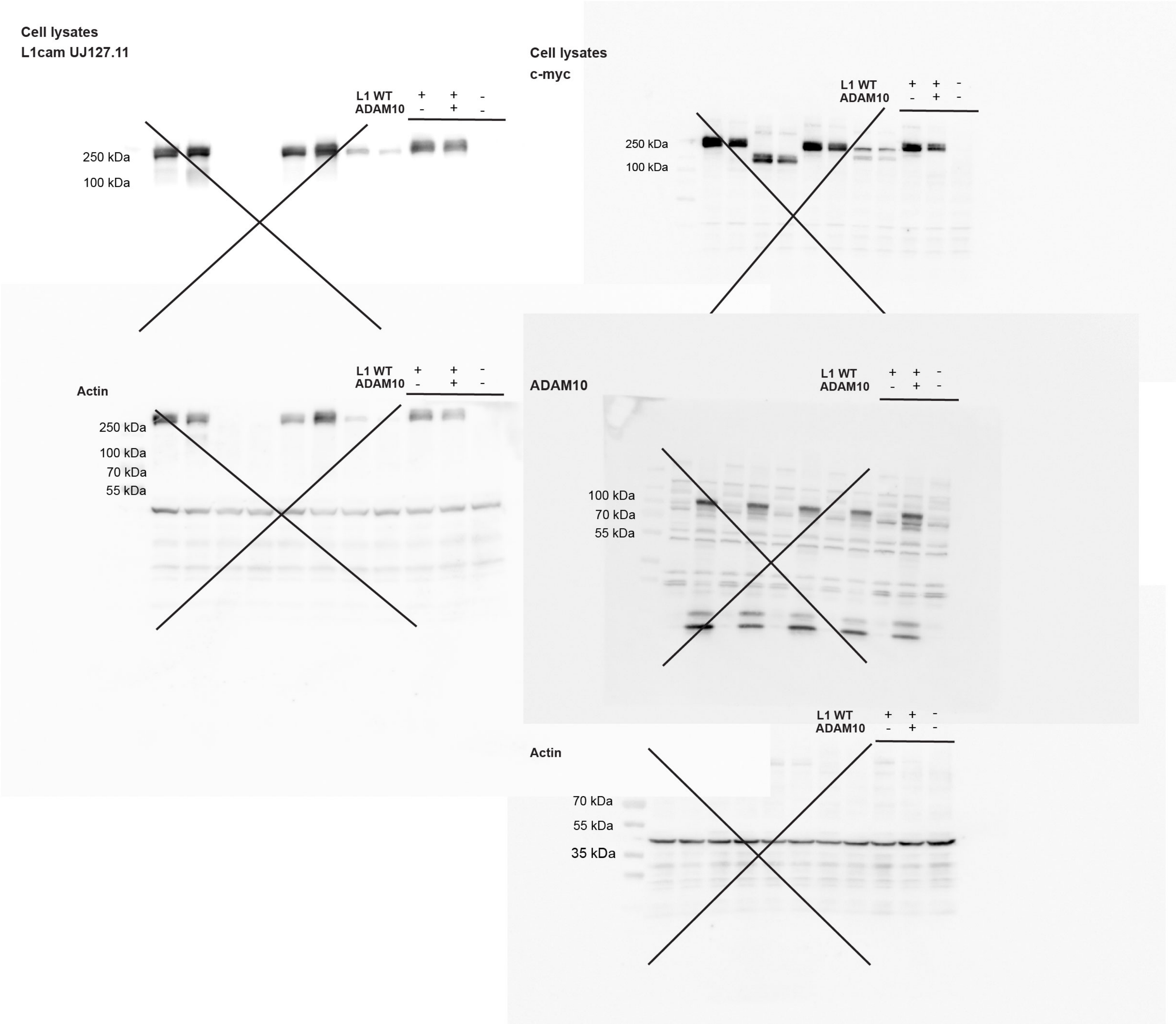

Supplement: Supplementary file 1 — Supplementary information [file 41598_2019_39884_MOESM1_ESM.pdf]
